# Tree-average distances on certain phylogenetic networks have their weights uniquely determined

**DOI:** 10.1186/1748-7188-7-13

**Published:** 2012-05-15

**Authors:** Stephen J Willson

**Affiliations:** 1 Department of Mathematics, Iowa State University, Ames, IA 50011 USA

**Keywords:** digraph, distance, metric, hybrid, network, tree-child, normal network, phylogeny

## Abstract

A phylogenetic network *N *has vertices corresponding to species and arcs corresponding to direct genetic inheritance from the species at the tail to the species at the head. Measurements of DNA are often made on species in the leaf set, and one seeks to infer properties of the network, possibly including the graph itself. In the case of phylogenetic trees, distances between extant species are frequently used to infer the phylogenetic trees by methods such as neighbor-joining.

This paper proposes a *tree-average *distance for networks more general than trees. The notion requires a *weight *on each arc measuring the genetic change along the arc. For each displayed tree the distance between two leaves is the sum of the weights along the path joining them. At a hybrid vertex, each character is inherited from one of its parents. We will assume that for each hybrid there is a probability that the inheritance of a character is from a specified parent. Assume that the inheritance events at different hybrids are independent. Then for each displayed tree there will be a probability that the inheritance of a given character follows the tree; this probability may be interpreted as the probability of the tree. The *tree-average *distance between the leaves is defined to be the expected value of their distance in the displayed trees.

For a class of rooted networks that includes rooted trees, it is shown that the weights and the probabilities at each hybrid vertex can be calculated given the network and the tree-average distances between the leaves. Hence these weights and probabilities are uniquely determined. The hypotheses on the networks include that hybrid vertices have indegree exactly 2 and that vertices that are not leaves have a tree-child.

## 1 Introduction

In phylogeny, the evolution of a collection of species is modelled via a directed graph in which the vertices are species and the arcs indicate direct descent, usually with modification as mutations accumulate. The leaves typically correspond to extant species, while internal vertices typically correspond to presumed ancestors. It has been common to assume that the directed graphs are trees, but more recently more general networks have also been studied so as to include the possibility of hybridization of species or lateral gene transfer. General frameworks for phylogenetic networks are discussed in [1], [2], [3], and [4]. See also the recent book [5].

There are many methods to reconstruct phylogenetic trees from information such as the DNA of extant species. The most generally accepted methods include maximum parsimony, maximum likelihood, and Bayesian. See [6] for an overview. These methods, however, are only heuristic, do not guarantee an optimal solution, and can be very time-consuming for a moderate number of species.

Suppose *X *denotes the set of extant species for some analysis, including an outgroup which is used to locate the root. The DNA information may be summarized via the computation of distances between members of *X*. If *x*, *y *∈ *X*, then *d*(*x*, *y*) summarizes the amount of genetic difference between the DNA strings of *x *and *y*. In order to compensate at least partially for the possibility of repeated mutation at the same site, a number of different distances are in use, based on different models of mutation. Notable examples include the Jukes-Cantor [7], Kimura [8], HKY [9], and log determinant [10], [11] distances. The log determinant distance is especially interesting in that it can be proved that typically the distances add along the paths, so that the distance along a path is the sum of the distances for each edge along the path.

Some fast methods to reconstruct phylogenetic trees make use of distances between members of *X*. Probably the most common distance-based method is Neighbor-joining [12]. It is computationally fast. It often gives a good initial tree with which heuristic methods begin in order to find an improved tree by other methods. Another more recent method FastME [13], [14] is based on the principle of balanced minimum evolution, in which one assumes that the correct tree is the one that exhibits the minimal total amount of evolution, suitably measured.

Distance-based methods have been rarely used to construct phylogenetic networks that are not necessarily trees. It is true that distances occur in common exploratory methods to display the diversity of trees for the same species such as the split decomposition (see [15] or an overview in [5]). These distances, however, are not derived from any biologically based model of evolution.

**Figure 1:**
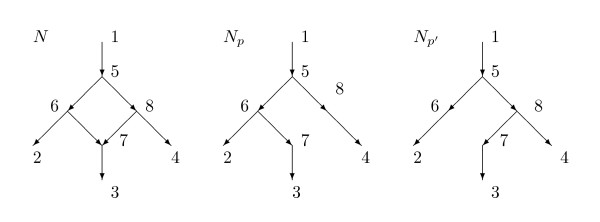
**A network *N *with root 1, and the two trees *N_p _*and *N_p_*_' _that it displays**. If *N *is equiprobable, then 7 inherits approximately half its characters from 6 and the other characters from 8.

This paper studies a distance on rooted directed networks that is based upon a model of evolution. Consider, for example, the network *N *in Figure 1. The root is 1 and there is a hybridization event at 7 with parents 6 and 8. Vertex 7 is called a *hybrid *vertex or a *reticulation *vertex. For some characters, the character state at 7 is inherited from the parental species 6, while for other characters the character state at 7 is inherited from species 8. For character states inherited from 6 the evolutionary history is best described by the displayed tree *N_p_*, while for character states inherited from 8 the history is best described by the tree *N_p_*_'_. Here *p *and *p*' are *parent maps *telling the parent of every non-root vertex. In the example *p*(7) = 6 while *p*'(7) = 8. Each parent map *p *leads to a displayed tree *N_p_*.

In Figure 1, each arc might have a numerical *weight *measuring the amount of genetic change on the arc. In either tree *N_p _*or *N_p_*_' _the distance between two vertices might be plausibly defined as the sum of the weights of the edges on the unique path between the vertices. This paper explores the possibility that an appropriate distance between the vertices in the network *N *is a weighted average of the distances in *N_p _*and *N_p_*_'_.

More generally, the trees displayed by a network *N *will be conveniently indexed as *N_p _*where *p *ranges over all the parent maps. Let *Par*(*N*) denote the set of all parent maps for *N*. For each hybrid vertex *h*, the probability that a character of *h *is inherited from a particular parent vertex *q_i _*will be denoted *α*(*q_i_*, *h*). Assume that these inheritances at different hybrid vertices are independent events. Then for each *p *∈ *Par*(*N*) we obtain that the probability *Pr*(*p*) that the tree *N_p _*models the inheritance of a particular character is given by

Pr(p)= ∏[α(p(h),h):h is hybrid].

If *x *and *y *are vertices, then the distance between *x *and *y *in *N_p_*, written *d*(*x*, *y*; *N_p_*), is the sum of the weights of arcs on the unique path joining *x *and *y *in *N_p_*. The *tree-average distance d*(*x*, *y*; *N*) between *x *and *y *in *N *will be defined to be the expected value of the distances in the various trees *N_p_*:

$$d\left(x,y;N\right)=\sum \left[Pr\left(p\right)d\left(x,y;N_{p}\right):p\in Par\left(N\right)\right].$$

If a hybrid vertex *h *satisfies that each parent *q *of *h *has the same probability, we will call the inheritance *equiprobable at h*. This special case assumes that the contribution from each parent to *h *is the same; if there are two parents, each contributes approximately 50%.

In Figure 1 note that, for each species in the leafset *X *= {1, 2, 3, 4}, it is plausible that the DNA is available since 2, 3, 4 correspond to extant species and 1 to an extant outgroup species. Hence it is plausible that we know *d*(*x*, *y*; *N*) for distinct *x *and *y *in *X*, hence $\left(\begin{array}{c} 4\\ 2 \end{array}\right)=6$ nonzero distances. Nevertheless, *N *has 8 arcs and hence it is not likely that from the 6 known distances we could compute 8 independent weights for these arcs. Indeed, the equations obtained in this paper for this network have infinitely many solutions. There is a possibility of simultaneous identical mutations between 6 and 7 and between 8 and 7 which might be confused with mutations between 7 and 3.

In this paper we will assume that the weight of an arc into a hybrid vertex is 0. Thus in Figure 1, the weights of arcs (6, 7) and (8, 7) will be zero. Under this assumption vertex 7 corresponds roughly to the immediate offspring of a hybridization event, in which some characters came intact from 6 and the remainder intact from 8. Further mutation occurred before species 3 evolved from 7.

Note that the number of arcs of *N *in Figure 1 that are not directed into a hybrid vertex is 6. It is therefore plausible that given the 6 numbers *d*(*x*, *y*; *N*) for *x*, *y *∈ {1, 2, 3, 4}, we might be able to recover the weights for each of the 6 arcs in *N *that are not directed into the hybrid vertex 7. These same weights would be utilized in distances for both *N_p _*and *N_p_*_'_. On the other hand, we should like to determine an additional parameter *α*(6, 7) telling the probability of inheritance by 7 of a character from 6. It is unlikely that six equations, one for each *d*(*x*, *y*; *N*), will uniquely and generically determine seven real parameters. Indeed, the methods of this paper for this example lead to six equations in seven unknowns such that for certain values of the distances the weights and probabilities are not uniquely determined. Consequently for the situation in Figure 1 we will assume that *α*(6, 7) = *α*(8, 4) = 1/2; we call the inheritance *equiprobable at 7*.

**Figure 2:**
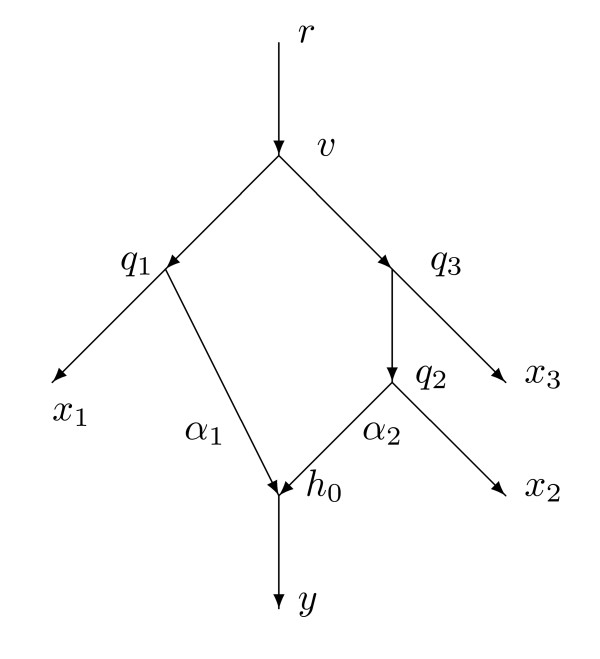
**A minimal configuration needed to be able to find the probability *α*_1 _= *α*(*q*_1_, *h*_0_) = 1 *- α*_2 _that a character state in *h*_0 _is inherited from *q*_1_**.

By contrast, Figure 2 shows another network with *X *= {*r*, *x*_1_, *x*_2_, *x*_3_, *y*} containing a single hybrid vertex *h*_0_. In this case there are $\left(\begin{array}{c} 5\\ 2 \end{array}\right)=10$ distances and 8 arcs not into a hybrid vertex, so it is plausible that the 10 equations would allow us to uniquely determine a ninth parameter *α*_1 _= *α*(*q*_1_, *h*_0_) satisfying 0 *< α*_1 _*<*1. In fact, this paper will show how to determine all 9 parameters. Then *α*(*q*_2_, *h*_0_) = 1 - *α*_1 _is also determined. In Figure 2 we will not need to assume equiprobability at *h*_0_.

**Figure 3:**
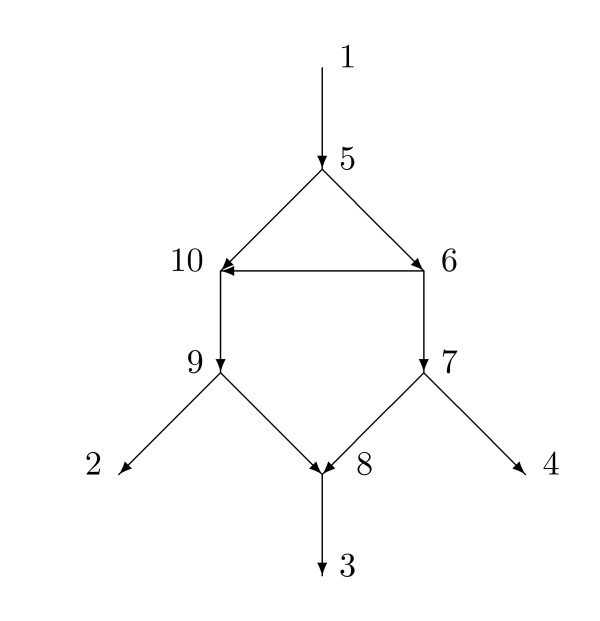
**This tree-child network has *X *= {1, 2, 3, 4}**. There are 7 arcs not leading to a hybrid vertex but only 6 distances, and the weights are not uniquely determined. The network is not normal because arc (5, 10) is redundant.

In order to obtain interesting results, assumptions must be made about the network *N*. As an extreme case it would be easy to add many more internal vertices and edges to the network *N *of Figure 1 without adding any additional leaves yet increasing arbitrarily the number of arcs. For example, Figure 3 shows a network in which the network *N *of Figure 1 has been modified by the addition of other arcs. The 6 distances do not determine the weights for all 7 arcs that do not lead to a hybrid vertex in Figure 3.

Particular kinds of acyclic networks have been studied in various papers. Wang *et al*. [16] and Gusfield *et al*. [17] study "galled trees" in which all recombination events are associated with node-disjoint recombination cycles; the idea occurs also earlier in [18]. Choy *et al*. [19] and Van Iersel *et al*. [20] generalized galled trees to "level-*k*" networks. Baroni, Semple, and Steel [2] introduced the idea of a "regular" network, which coincides with its cover digraph. Cardona *et al*. [21] discussed "tree-child" networks, in which every vertex not a leaf has a child that is not a reticulation vertex. An arc (*a*, *b*) is *redundant *if there is a directed path from *a *to *b *that that does not utilize this arc. The current author has utilized "normal" networks [22] which are both tree-child and contain no redundant arc.

Most results in this paper assume that the network is *normal*. This means, briefly, that every vertex not in *X *and not a leaf has a tree-child (a child with indegree one); and moreover, there is no redundant arc. For example, if *X *= {1, 2, 3, 4} then the network in Figure 1 is normal while the network in Figure 3 is not normal since arc (5,10) is redundant. With the assumption that there are no redundant arcs we show in Section 3 that for a given network *N*, the tree-average distance *d *is a metric on *X*. With the assumption of normality we also show that different parent maps *p *yield different displayed trees *N_p_*. Hence the average over the parent maps *p *is the same as the average over displayed trees. This result eliminates the logical possibility that different parent maps *p*_1 _and *p*_2 _might yield displayed trees that are topologically the same, yielding an uncertainty about which is the correct average to use in the definition.

The main result, Theorem 4.1, assumes that the network *N *is normal and also that for all hybrid vertices the indegree is exactly 2 and the outdegree is exactly 1. At each hybrid vertex *h *we assume either equiprobability or else that *h *has a grandparent on at least one side of the reticulation cycle, as in Figure 2 but not Figure 1. Then from knowledge both of *N *and of the tree-average distance function *d*, the weights for all arcs are uniquely determined and indeed can be computed by explicit formulas. Moreover, the probabilities of inheritance at each hybrid vertex are uniquely determined and can be computed by explicit formulas. This calculation is, of course, trivial if the network is equiprobable at *h*.

A model for a distance function containing certain parameters is called *identifiable *if the parameters can be reconstructed from the (exact) values of the distance function. Theorem 4.1 thus asserts that, if the tree-average distance function *d *on *X *and the network *N *are known, then the real parameters of the model (i.e., the weights and the probabilities) are identifiable in various cases.

A major problem, of course, is the reconstruction of *N *itself from a distance function *d*. I have obtained partial results (not included in this paper) which give a reconstruction of *N *itself when the distance *d *is the tree-average distance and when the network *N *satisfies the hypotheses of Theorem 4.1 and some additional hypotheses. The reconstruction of *N *is possible because of the simple forms of the formulas obtained in this paper. Essentially, the formulas are simple enough that they can be used recursively when only part of the network is yet known. I plan a subsequent paper which will utilize the results in the current paper to reconstruct *N *from the tree-average distances.

The assumption that all hybrid vertices have indegree 2, assumed in Theorem 4.1, is plausible biologically since in sexually reproducing species an offspring arises from one egg and one sperm.

The assumption that there be no redundant arcs is essential for Theorem 4.1. Figure 3 displays a tree-child network *N *with *X *= {1, 2, 3, 4}. There are 6 independent nonzero distances between the members of *X*, yet there are 7 arcs not directed into hybrid vertices. It is easy to choose positive values for the tree-average distances such that there are infinitely many positive choices of the weights given the network. Note that each vertex not a leaf has a tree-child, so the network is a tree-child network [21]. Hence Theorem 4.1 cannot be extended to general tree-child networks.

Some other extensions of the current results and problems are discussed in the concluding section 6.

## 2 Fundamental Concepts

A *directed graph *or *digraph *(*V*, *A*) consists of a finite set *V *of *vertices *and a finite set *A *of *arcs*, each consisting of an ordered pair (*u*, *v*) where *u *∈ *V *, *v *∈ *V *, *u *≠ *v*. We interpret (*u*, *v*) as an arrow from *u *to *v *and say that the arc *starts *at *u *and *ends *at *v*. There are no multiple arcs and no loops. If (*u*, *v*) ∈ *A*, say that *u *is a *parent *of *v *and *v *is a *child *of *u*. A *directed path *is a sequence *u*_0_, *u*_1_, ..., *u_k _*of vertices such that for *i *= 1, ..., *k*, (*u*_i - 1_, *u_i_*) ∈ *A*. The path is *trivial *if *k *= 0. Write *u *≤ *v *if there is a directed path starting at *u *and ending at *v*. The digraph is *acyclic *if there is no nontrivial directed path starting and ending at the same point. If the digraph is acyclic, it is easy to see that ≤ is a partial order on *V *.

The *indegree *of vertex *u *is the number of *v *∈ *V *such that (*v*, *u*) ∈ *A*. The *outdegree *of *u *is the number of *v *∈ *V *such that (*u*, *v*) ∈ *A*. A *leaf *is a vertex of outdegree 0. A *normal vertex *(or *tree vertex *) is a vertex of indegree 1. A *hybrid *vertex (or *reticulation vertex *) is a vertex of indegree at least 2. An arc (*u*, *v*) is a *normal arc *if *v *is a normal vertex.

A digraph (*V*, *A*) is *rooted *if it has a unique vertex *r *∈ *V *with indegree 0 such that, for all *v *∈ *V *, *r *≤ *v*. This vertex *r *is called the *root*.

Let *X *denote a finite set. Typically in phylogeny, *X *is a collection of species. Measurements are assumed to be possible among members of *X*, so that we may assume that, for example, their DNA is known for each *x *∈ *X*.

A *phylogenetic X-network N *= (*V*, *A*, *r*, *X*) is a rooted acyclic digraph *G *= (*V*, *A*) with root *r *such that there is a one-to-one map *ϕ *: *X *→ *V *whose image contains all vertices *v *such that either

(i) *v *is a leaf; or

(ii) *v *= *r*; or

(iii) *v *has indegree 1 and outdegree 1.

There may be additional vertices in *X*. We will identify each *x *∈ *X *with its image *ϕ*(*x*). The set *X *will be called the *base-set *for *N*.

In biology the network gives a hypothesized relationship among the members of *X*. It is quite common also that a certain extant *outgroup *species *r*' is assumed to have evolved separately from the rest of the species in question. When this happens, we identify the species *r*' with the root *r*. Thus extant species (the leaves) are in *X *by (i) since measurements can be made on them. The outgroup *r*', which is identified with the root, is in *X *by (ii). If a vertex has indegree 1 and outdegree 1 then nothing uniquely determines it unless, for fortuitous reasons, it is possible to make measurements on its DNA, in which case it lies in the base-set *X*.

An *X-tree *is a phylogenetic *X*-network such that the underlying digraph is a tree.

**Figure 4:**
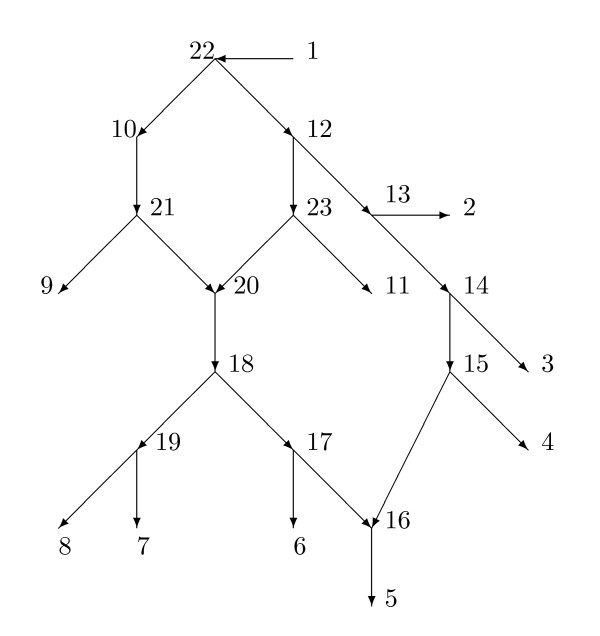
**A normal phylogenetic *X*-network with *X *= {1, 2, 3, 4, 5, 6, 7, 8, 9, 10, 11} and root 1**. The root corresponds to an outgroup species. Measurements on DNA are assumed possible on members of *X*.

Figure 4 shows a phylogenetic *X*-network *N *with base-set *X *= {1, 2, 3, 4, 5, 6, 7, 8, 9, 10, 11}. The root is *r *= 1. Note that the leaves are in *X *by (i), 1 ∈ *X *by (ii), and 10 ∈ *X *by (iii). Measurements such as DNA are assumed possible on members of *X*. Since the root 1 is actually an outgroup and the leaves are all extant, this is plausible for all members of *X *except 10. We are perhaps here assuming that, by some fortuitous chance, some historical DNA of 10 is also available.

An arc (*u*, *v*) ∈ *A *is *redundant *if there exists *w *∈ *V *such that *u*, *v*, and *w *are distinct and *u *≤ *w *≤ *v*. The removal of a redundant arc (*u*, *v*) still leaves *u *≤ *v *in the network.

A phylogenetic *X*-network *N *= (*V*, *A*, *r*, *X*) with base-set *X *is *normal *provided (1) whenever *v *∈ *V *and *v *∉ *X*, then *v *has a tree-child *c*; and (2) there are no redundant arcs. The networks in Figure 2 and 4 are normal, while the network of Figure 3 is not normal. The usage here of "normal" differs slightly from that in [22] in that here hybrid vertices that are not leaves may have outdegree 1, whereas in [22] hybrid vertices that were not leaves had outdegree 2 or higher. There is an obvious one-to-one relationship between normal networks in the current sense and normal networks in the previous sense.

A normal network *N *is *semibinary *if each hybrid node has indegree 2 and outdegree 1. It follows from normality that the child of the hybrid node is necessarily normal.

A *normal path *in *N *from *v *to *x *is a directed path *v *= *v*_0_, *v*_1_, ..., *v_k _*= *x *such that for *i *= 1, ... *k*, *v_i _*is normal. A *normal path from v to X *is a normal path starting at *v *and ending at some *x *∈ *X*. For example, in Figure 4, the path 20, 18, 19, 8 is normal and is a normal path from 20 to *X*. The path 18, 17, 16, 5 is not normal since 16 is hybrid. The trivial path 3 is normal.

Suppose *N *is normal and *v *∈ *V *. Then there is a normal path from *v *to *X*. To see this, if *v *∈ *X*, then the trivial path is a normal path from *v *to *X*. If *v *∉ *X*, then *v *has a tree child *v*_1_. If *v*_1 _∈ *X*, then the path *v*_0_, *v*_1 _is a normal path to *v*_1 _in *X*. Otherwise *v*_1 _has a tree-child *v*_2_. If *v*_2 _∈ *X *then the path *v*_0_, *v*_1_, *v*_2 _is a normal path from *v *to *v*_2 _in *X*. Proceeding in this manner, we obtain the result.

Suppose two normal paths shared a common vertex *x*, say the normal paths *v *= *v*_0_, ..., *v_k _*= *x *and *w *= *w*_0_, ..., *w_j _*= *x*. If *k >*0 and *j >*0 then since *x *is normal with a unique parent, it follows that *v_k - _*_1 _= *w_j - _*_1_. Repeating the argument we find that either there is an *i *such that *v *= *w_i _*or else there is an *i *such that *w *= *v_i_*. This argument, of frequent use, is called *following the normal paths backwards*.

A *graph *(or, for emphasis, an *undirected graph*) (*V*, *E*) consists of a finite set *V *of *vertices *and a finite set *E *of *edges*, each a subset {*v*_1_, *v*_2_} of *V *consisting of two distinct vertices. Thus an edge has no direction, while an arc has a direction. If *N *= (*V*, *A*, *r*, *X*) is a phylogenetic *X*-network, there is an associated undirected graph *Und*(*N*) = (*V*, *E*) in which every arc in *A *has its direction ignored; thus *E *= {{*a*, *b*}: (*a*, *b*) ∈ *A *or (*b*, *a*) ∈ *A*}.

## 3 The Tree-Average Distance

If *N *= (*V*, *A*, *r*, *X*) is a phylogenetic *X*-network, then a *parent map p *for *N *consists of a map *p *: *V *-{*r*} → *V *such that, for all *v *∈ *V *- {*r*}, *p*(*v*) is a parent of *v*. Note that *r *has no parent. If *v *is normal, then there is only one possibility for *p*(*v*), while if *v *is hybrid, there are at least two possibilities for *p*(*v*). In Figure 4, an example of a parent map *p *satisfies *p*(20) = 23, *p*(16) = 17, and for all other vertices *v *besides 1, *p*(*v*) is the unique parent of *v*.

Write *Par *(*N*) for the set of all parent maps for *N*. In general if there are *k *distinct hybrid vertices and they have indegrees respectively *i*_1_, *i*_2_, ..., *i_k_*, then the number of distinct parent maps *p *is |*Par *(*N*)| = ∏[*i_j _*: *j *= 1, ..., *k*]. If *N *is a network with *k *distinct hybrid vertices, each of indegree 2, then |*Par*(*N*)| = 2*^k^*.

Given *p *∈ *Par *(*N*) the set *A_p _*of *p-arcs *is *A_p _*= {(*p*(*v*), *v*): *v *∈ *V *- {*r*}}. The *induced tree N_p _*is the directed graph (*V*, *A_p_*) with root *r*. Note that each vertex in *V *- {*r*} has a unique parent in *N_p_*. Thus *N_p _*is a tree with vertex set *V *. The set *X*, however, need not be a base-set of *N_p_*. For example, if *h *is hybrid in *N*, then in *N_p _*the vertex *h *has indegree 1 from the arc (*p*(*h*), *h*) and outdegree 1, yet need not lie in *X*.

Several of the proofs will require the notion of "complementary parents". Suppose *p *∈ *Par *(*N*) and *h *is a particular hybrid vertex with exactly two parents *q*_1 _and *q*_2_. Assume *p*(*h*) = *q*_1_. The *complementary parent map p*' of *p with respect to h *is defined by

$$p^{\prime }\left(v\right)=\left\{\begin{array}{cc} p\left(v\right) & \text{if}\;v\neq h\\ q_{2} & \text{if}\;v=h. \end{array}\right)$$

Thus *p*' agrees with *p *except at *h*, where *p*' chooses the other parent from that chosen by *p*.

A phylogenetic *X*-network is *weighted *provided that for each arc (*a*, *b*) ∈ *A *there is a non-negative number *ω*(*a*, *b*) called the *weight of *(*a*, *b*) such that

(1) if *b *is hybrid, then *ω*(*a*, *b*) = 0;

(2) if *b *is normal, then *ω*(*a*, *b*) ≥ 0.

We call the function *ω *from the set of arcs to the reals the *weight function *of *N*. We interpret *ω *(*a*, *b*) as a measure of the amount of genetic change from species *a *to species *b*. If *h *is hybrid with parents *q*_1 _and *q*_2 _and unique child *c*, then the hybridization event is essentially assumed to be instantaneous between *q*_1 _and *q*_2 _with no genetic change in those character states inherited by *h *from *q*_1 _or *q*_2 _respectively. Further mutation then occurs from *h *to *c*, as measured by *ω *(*h*, *c*).

In any rooted tree *T *= (*V*, *A*, *r*), two vertices *u *and *v *have a unique *most recent common ancestor *mrca(*u*, *v*) = mrca(*u*, *v*; *T *) ∈ *V *that satisfies

(1) mrca(*u*, *v*) ≤ *u *and mrca(*u*, *v*) ≤ *v*;

(2) whenever *z *≤ *u *and *z *≤ *v*, then *z *≤ mrca(*u*, *v*).

In a network that is not a tree, two vertices *u *and *v *need not have a mrca(*u*, *v*).

Suppose that *N *= (*V*, *A*, *r*, *X*) is a weighted phylogenetic *X*-network with weight function *ω*. For each *p *∈ *Par *(*N*) and for each *u*, *v *∈ *V *, define the distance *d*(*u*, *v*; *N_p_*) as follows: in *N_p _*there is a unique undirected path *P *(*u*, *v*) between *u *and *v*; defined (*u*, *v*; *N_p_*) to be the sum of the weights of arcs along *P *(*u*, *v*). More precisely, since *N_p _*is a tree, there exists a most recent common ancestor *m *= mrca(*u*, *v*; *N_p_*), a directed path *P*_1 _given by *m *= *u*_0_, *u*_1_, . . ., *u_k _*= *u *from *m *to *u*, and a directed path *P*_2 _given by *m *= *v*_0_, *v*_1_, . . ., *v_j _*= *v *from *m *to *v*. Define

$$d\left(u,v;N_{p}\right)=\sum \left[\omega \left(u_{i},u_{i+1}\right):i=0,\cdots \;,k-1\right]+\sum \left[\omega \left(v_{i},v_{i+1}\right):i=0,\cdots \;,j-1\right].$$

We shall refer to *d*(*u*, *v*; *N_p_*) as the *distance between u and v in N_p_*.

Let *H *denote the set of hybrid vertices of *N*. For each *h *∈ *H*, let *P *(*h*) denote the set of parents of *h*, i.e. the set of vertices *u *such that (*u*, *h*) ∈ *A*. Since *h *∈ *H*,*|P *(*h*)| *≥ *2. For each *u *∈ *P *(*h*), let *α*(*u*, *h*) denote the fraction of the genome that *h *inherits from *u*. We may interpret α(*u*, *h*) as the probability that a character is inherited by *h *from *u*, so for all *h *∈ *H*, ∑[*α*(*u*, *h*): *u *∈ *P*(*h*)] = 1.

If *h *and *h*' are distinct members of *H*, we will assume that the inheritances at *h *and *h*' are independent. More generally, suppose for every *h *∈ *H *that *q_h _*is a parent of *h*. Then we assume that the events that a character at *h *is inherited from *q_h _*are independent. It is then easy to see that for each *p *∈ *Par*(*N*) the probability that inheritance follows the parent map *p *is *Pr *(*p*) = ∏[*α*(*p*(*h*), *h*): *h *∈ *H*].

The *tree-average distance d*(*u*, *v*; *N*) between *u *and *v *in *N *is defined by

$$d\left(u,v;N\right)=\sum \left[Pr\left(p\right)d\left(u,v;N_{p}\right):p\in Par\left(N\right)\right].$$

It is thus the expected value of the distances between *u *and *v *in the various *N_p_*.

The simplest situation has each parent of *h *equally likely, so *α*(*p*(*h*), *h*) = 1/|*P *(*h*)| for each *p *∈ *Par*(*N*). If this situation occurs, we call the network *equiprobable at h*. If the network *N *is equiprobable at *h *for all *h *∈ *H*, then we call the network *equiprobable*, and for each *u *and *v *in *X*, *d*(*u*, *v*; *N*) is the average of the values *d*(*u*, *v*; *N_p_*) for *p *∈ *Par*(*N*).

For example, for the network *N *in Figure 1 suppose that the arcs have weights given by *ω*(1, 5) = 1 = *ω*(5, 6) = *ω*(7, 3), while *ω*(5, 8) = *ω*(8, 4) = 2 and *ω*(6, 2) = 4. Since 7 is hybrid, *ω*(6, 7) = *ω*(8, 7) = 0. Suppose, as in Figure 1, the parent map *p *satisfies *p*(7) = 6 while the parent map *p*' satisfies *p*' (7) = 8. Then *N_p _*shown in Figure 1 is obtained from *N *by deleting the arc (8, 7) while *N_p_*_' _is obtained from *N *by deleting the arc (6, 7). Assume *α*(6, 7) = 1/3 and *α*(8, 7) = 2/3, so *Pr*(*p*) = 1/3, *Pr*(*p*') = 2/3. To compute *α*(1, 3; *N*) we find *d*(1, 3; *N_p_*) = *ω*(1, 5) + *ω*(5, 6) + *ω*(6, 7) + *ω*(7, 3) = 1 + 1 + 0 + 1 = 3, *d*(1, 3; *N_p_*_'_) = *ω*(1, 5) + *ω*(5, 8) + *ω*(8, 7) + *ω*(7, 3) = 1 + 2 + 0 + 1 = 4. Hence *d*(1, 3; *N*) = (1/3)*d*(1, 3; *N_p_*) + (2/3)*d*(1, 3; *N_p_*_'_) = (1/3)(3) + (2/3)(4) = 11/3. For another example *d*(1, 2; *N_p_*) = *d*(1, 2; *N_p_*_'_) = 6 so *d*(1, 2; *N*) = (1/3)(6) + (2/3)(6) = 6.

Given *u *and *v*, the vertices mrca(*u*, *v*; *N_p_*) may differ for different *p*. This is seen in Figure 1 where mrca(2, 3; *N_p_*) = 6 while mrca(2, 3; *N_p_*_'_) = 5.

**Theorem 3.1**. *Assume N *= (*V*, *A*, *r*, *X*) *is a phylogenetic X-network that has no redundant arcs. Assume N has a weight function ω satisfying that ω*(*a*, *b*) *>*0 *if b is normal. Then the tree-average distance on X from N is a metric on X*.

*Proof*. A metric *d *on *X *must satisfy

(1) For all *x *and *y *in *X*, *d*(*x*, *y*) ≥ 0 and *d*(*x*, *y*) = 0 iff *x *= *y*.

(2) For all *x *and *y *in *X*, *d*(*x*, *y*) = *d*(*y*, *x*).

(3) For all *x*, *y*, *z *∈ *X*, *d*(*x*, *z*) ≤ *d*(*x*, *y*) + *d*(*y*, *z*).

For (2), suppose *x*, *y*, ∈ *X*. For all *p*, *d*(*x*, *y*; *N_p_*) = *d*(*y*, *x*; *N_p_*), whence *d*(*x*, *y*; *N*) = *d*(*y*, *x*; *N*).

For (3) suppose *x*, *y*, *z *∈ *X*. For each *N_p_*, *d*(*x*, *z*; *N_p_*) ≤ *d*(*x*, *y*; *N_p_*) + *d*(*y*, *z*; *N_p_*) from the truth of the four-point condition, see [23], p 147. Hence the result follows for distances in *N *as well.

For (1) it is clear that for each *p*, *d*(*x*, *y*; *N_p_*) ≥ 0, whence *d*(*x*, *y*; *N*) ≥ 0. Moreover, for each *p*, *d*(*x*, *x*; *N_p_*) = 0, whence *d*(*x*, *x*; *N*) = 0.

To finish the proof of (1), suppose *d*(*x*, *y*; *N*) = 0; we show *x *= *y*. Assume instead *x *≠ *y*. Since the weights are nonnegative, for every *p *we have *d*(*x*, *y*; *N_p_*) = 0. Hence for every *p *∈ *Par*(*N*), in *N_p _*the unique path between *x *and *y *contains only arcs (*a*, *b*) with *b *hybrid in *N*.

If *x *and *y *are both normal, then for every *p *the unique path between *x *and *y *in *N_p _*must consist of a directed path from *v *= mrca(*x*, *y*; *N_p_*) to *x *and a path from *v *to *y*; hence it contains a normal arc whence *d*(*x*, *y*; *N_p_*) *>*0. Thus we may assume that one vertex, say *y*, is hybrid.

In *N *choose a directed path *P *= *y*_0_, *y*_1_, ..., *y_k _*= *y *such that *y*_1 _is not hybrid but *y*_2_, ..., *y_k _*are hybrid. This is always possible because there is a directed path from *r *to *y*, say *u*_0 _= *r*, *u*_1_, *u*_2_, ..., *u_k _*= *y*. The child *u*_1 _of *r *cannot be hybrid, because if it were, then its other parent *q *besides *r *must also have a path to *q *from *r*, and this path combined with the arc (*q*, *u*_1_) would make the arc (*r*, *u*_1_) redundant. Moreover, we may choose this path so that *x *does not lie in {*y*_1_, ..., *y_k_*} since whenever *y_i _*is hybrid there are at least two choices of the parent *y_i - _*_1_, and we may select *y_i - _*_1 _to be distinct from *x*.

If *x *is normal in *N*, let *Q *be the trivial path *z*_0 _= *x*. Otherwise we may choose a directed path *Q *= *z*_0_, *z*_1_, ..., *z_s _*= *x *such that *z*_0 _is not hybrid but all other vertices are hybrid. Moreover, we may assume that the vertices of *Q *are all distinct from the vertices of *P *. This is because, if *z_i _*is hybrid, it cannot have two parents *q*_1 _and *q*_2 _which are on *P *since then there must be a directed path from say *q*_1 _to *q*_2_, whence the arc (*q*_1_, *z_i_*) is redundant.

Since the vertices on *P *and *Q *are distinct, there exists a parent map *p *that agrees with all the choices made in constructing both *P *and *Q*. Hence in *N_p_*, *P *is a path from *y*_0 _to *y*, *Q *is a path form *z*_0 _to *x*, and the paths are disjoint. In *N_p _*let *v *= mrca(*y*_0_, *z*_0_; *N_p_*). Then in *N_p _*the unique path between *x *and *y *consists of *P *, *Q*, a path from *v *to *y*_0_, and a path from *v *to *z*_0_. Since *y*_1 _and *z*_0 _are normal, this path includes a normal arc, so *d*(*x*, *y*; *N_p_*) *>*0. It follows that *d*(*x*, *y*; *N*) *>*0, a contradiction. □

**Corollary 3.2**. *Assume N is a normal network with weight function ω such that ω*(*a*, *b*) *>*0 *if b is normal. Then the tree-average distance on X from N is a metric on X*.

The tree-average distance is defined as a weighted average in terms of parent maps. Any tree that arises as *N_p _*for some parent map *p *is said to be *displayed *in *N*. There is a logical possibility that several different parent maps *p *could yield essentially the same displayed tree. The next theorem gives sufficient conditions so that in fact the displayed trees are all distinct. Hence the tree-average distance becomes a weighted average over all the distinct displayed trees.

The proof requires the notion of a *split*. A *split of X *is a partition of *X *into exactly two nonempty subsets; if these are *A *and *B*, we write the split *A|B*. Two splits *A*_1_*|B*_1 _and *A*_2_*|B*_2 _are *compatible *if at least one of the sets *A*_1 _∩ *A*_2_, *A*_1 _∩ *B*_2_, *B*_1 _∩ *A*_2_, and *B*_1 _∩ *B*_2 _is the empty set. Removal of any edge *e *(but not its endpoints) from a tree *T *produces a split ∑(*e*) consisting of vertices in the connected components of *T *with *e *removed. The set of splits of a tree *T *will be denoted ∑(*T*). If *T *is directed, then the splits of *T *are obtained by reference only to the undirected tree so ∑(*T*) = ∑(*Und*(*T*)). By the Splits-Equivalence Theorem (see [23], p. 44) any two splits of a tree are compatible.

**Theorem 3.3**. *Assume N *= (*V*, *A*, *r*, *X*) *is a normal phylogenetic X-network*. *Suppose that every hybrid vertex that is not a leaf satisfies that it has outdegree 1 and that its unique child is normal. Suppose p and q are distinct parent maps for N. Then N_p _and N_q _are topologically distinct trees*.

*Proof*. We show that ∑(*N_p_*) and ∑(*N_q_*) are distinct. Since *p *≠ *q *there exists a hybrid vertex *h *such that *p*(*h*) ≠ *q*(*h*). Let *q*_1 _= *p*(*h*) and *q*_2 _= *q*(*h*). Choose a normal path in *N *from *q*_1 _to *x*_1 _∈ *X*, a normal path from *q*_2 _to *x*_2 _∈ *X*, and a normal path from *h *to *y *∈ *X*. Note that each normal path is a path in both *N_p _*and *N_q_*. Moreover, *q*_1 _is normal in *N *because otherwise its unique child would not be a tree-child. Similarly *q*_2 _is normal in *N*.

If ∑(*N_p_*) = ∑(*N_q_*), then each pair of splits would be compatible. In *N_p _*consider the split ∑(*a*, *q*_1_) where *a *is the unique parent of *q*_1 _and we remove the arc (*a*, *q*_1_) from *N_p_*. We may write ∑(*a*, *q*_1_) as *A*_1_*|B*_1 _where *A*_1 _contains *r*. The directed path in *N_p _*from *r *to *y *includes the arc (*a*, *q*_1_), so *B*_1 _contains *y*. Then *x*_1 _∈ *B*_1 _because there is a path from *q*_1 _to *x*_1 _and from *h *to *y*, neither of which includes (*a*, *q*_1_). Moreover, *x*_2 _∈ *A*_1_. To see this, since *N_p _*is rooted, there is a directed path from *r *to *q*_2_. If it included the arc (*a*, *q*_1_), then there would be a directed path in *N_p _*from *q*_1 _to *q*_2_; this is not possible since in that case the arc (*q*_1_, *h*) would be redundant in *N*, contradicting normality of *N*. Since *N_p _*contains the directed path from *q*_2 _to *x*_2 _missing the arc (*a*, *q*_1_), it follows that *x*_2 _∈ *A*_1_. Hence{*r*, *x*_2_} ⊆ *A*_1 _and {*y*, *x*_1_} ⊆ *B*_1_.

In *N_q _*consider the split ∑(*b*, *q*_2_) where *b *is the parent of *q*_2 _and we remove the arc (*b*, *q*_2_) from *N_q_*. Similarly to the case of *N_p _*we may write ∑(*b*, *q*_2_) = *A*_2 _| *B*_2 _where {*r*, *x*_1_} ⊆ *A*_2 _and {*y*, *x*_2_} ⊆ *B*_2_. If *N_p _*were topologically the same as *N_q_*, then these splits would need to be compatible. Yet *r *∈ *A*_1 _∩ *A*_2_, *x*_2 _∈ *A*_1 _∩ *B*_2_, *x*_1 _∈ *B*_1 _∩ *A*_2_, and *y *∈ *B*_1 _∩ *B*_2_, contradicting compatibility. □

**Corollary 3.4**. *Suppose N *= (*V*, *A*, *r*, *X*) *is a phylogenetic X-network that is normal. Suppose every hybrid vertex that is not a leaf has outdegree 1 and its unique child is normal. Suppose that there are exactly k hybrid vertices h*_1_*, h*_2_, ..., *h_k _and that for i *= 1, ..., *k, hybrid vertex h_i _has indegree d_i_. Then the total number of distinct trees displayed by N and the total number of parent maps are both *∏[*d_i _*: *i *= 1, ..., *k*].

## **4 Finding the weight function from ***d ***and ***N*

In this section we prove the main theorem, that the weights are determined by knowledge of *N *and the tree-average distances between members of *X*. For each hybrid vertex *h *we will assume either equiprobability at *h *or else a more complicated situation resembling Figure 2. The assumptions can be different at different hybrid vertices.

**Theorem 4.1**. *Suppose N *= (*V*, *A*, *r*, *X*) *is a phylogenetic X-network which is normal and semibinary. Let ω be a weight function on A satisfying ω*(*a*, *b*) = 0 *if b is hybrid and ω*(*a*, *b*) *≥ *0 *if b is normal. Assume that N is known and that the tree-average distance d*(*x*, *y*; *N*) *is known for each x and y in X*.

*For each hybrid vertex h with parents q*_1 _*and q*_2_*, assume either*

(1) the inheritance is equiprobable at h; or

*(2) at least one parent (say q*_2_*) satisfies that there exists q*_3 _*such that*

*(a) there is a normal path from q*_3 _*to q*_2_*;*

*(b) there is a normal path from q*_3 _*to some x*_3 _*in x which is disjoint from the normal path from q*_3 _*to q*_2 _*except for the vertex q*_3_*;*

*(c) there is no directed path from q*_3 _*to q*_1_ .

*Then the weight function ω is uniquely determined and can be computed explicitly. Moreover, for each hybrid h, the probabilities α*(*q_i_*, *h*) *for each parent q_i _of h are uniquely determined and can be computed explicitly*.

See Figure 2 to understand the assumptions about *h *in (2). Throughout this section we will assume the hypotheses of Theorem 4.1.

The proof primarily consists of a number of cases to handle different situations. We will present several of these special situations as lemmas and then later relate these together. Each lemma tells how certain distances or weights relate to distances between members of *X*.

**Lemma 4.2**. *Assume the hypotheses of Theorem 4.1. Suppose there is a normal path from a to b. Suppose there is a normal path from a to x *∈ *X which meets the normal path from a to b only in a. Suppose b has normal paths to y and z in X which are disjoint except at b. Then d*(*a*, *b*; *N*) = [*d*(*r*, *y*; *N*) + *d*(*x*, *z*; *N*) *- d*(*r*, *x*; *N*) *- d*(*y*, *z*; *N*)]/2.

**Figure 5:**
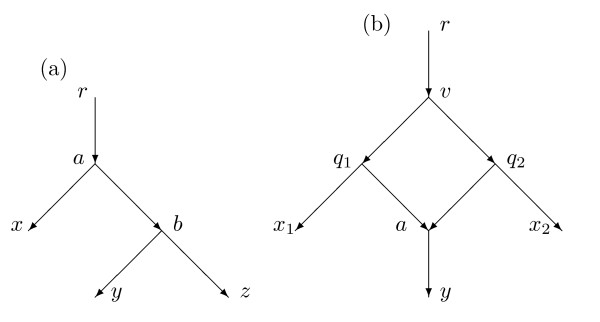
**The situation of Lemma 4.2 (a)**, **the situation of Lemmas 4.4 and 4.7 (b)**. If *p *is a parent map with *p*(*a*) = *q*_1_, the figure shows part of *N_p _*together with the arc (*q*_2_, *a*).

*Proof*. For each *p *∈ *Par*(*N*), the path from *a *to *b*, the path from *a *to *x*, the path from *b *to *y*, and the path from *b *to *z *must lie in *N_p _*since none of the arcs enters a hybrid vertex. Moreover, there must be a path from *r *to *a *which includes none of the arcs on the other paths mentioned above. See Figure 5a. Hence for each *p *∈ *Par*(*N*) one can verify

$$\begin{array}{c} d\left(r,y;N_{p}\right)=d\left(r,a;N_{p}\right)+d\left(a,b;N_{p}\right)+d\left(b,y;N_{p}\right)\\ d\left(x,z;N_{p}\right)=d\left(a,x;N_{p}\right)+d\left(a,b;N_{p}\right)+d\left(b,z;N_{p}\right)\\ d\left(r,x;N_{p}\right)=d\left(r,a;N_{p}\right)+d\left(a,x;N_{p}\right)\\ d\left(y,z;N_{p}\right)=d\left(b,y;N_{p}\right)+d\left(b,z;N_{p}\right). \end{array}$$

It follows that

$$\left[d\left(r,y;N_{p}\right)+d\left(x,z;N_{p}\right)-d\left(r,x;N_{p}\right)-d\left(y,z;N_{p}\right)\right]/2=d\left(a,b;N_{p}\right).$$

Taking expected values we see *d*(*a*, *b*; *N*) = ∑[*Pr*(*p*)*d*(*a*, *b*; *N_p_*): *p *∈ *Par*(*N*)] = ∑[*Pr*(*p*)[*d*(*r*, *y*; *N_p_*) + *d*(*x*, *z*; *N_p_*) *- d*(*r*, *x*; *N_p_*) *- d*(*y*, *z*; *N_p_*)]/2: *p *∈ *Par*(*N*)] = [*d*(*r*, *y*; *N*) + *d*(*x*, *z*; *N*) *- d*(*r*, *x*; *N*) *- d*(*y*; *z*; *N*)]/2. □

**Lemma 4.3**. *Assume the hypotheses of Theorem 4.1*.

*(1) Suppose *(*a*, *b*) *is an arc where a *∈ *X and b is normal. Suppose b has normal paths to y and z in X which are disjoint except at b. Then ω*(*a*, *b*) = [*d*(*a*, *y*; *N*) + *d*(*a*, *z*; *N*) - *d*(*y*, *z*; *N*)]/2.

*(2) Suppose there is a normal path from a to b *∈ *X. Suppose there is a normal path from a to x *∈ *X which intersects the path from a to b only in a. Then d*(*a*, *b*; *N*) = [*d*(*b*, *r*; *N*) + *d*(*b*, *x*; *N*) *- d*(*r*, *x*; *N*)]/2.

*In particular, suppose *(*a*, *b*) *is an arc, b *∈ *X is normal, and there is normal path from a to x *∈ *X which does not include b. Then ω*(*a*, *b*) = [*d*(*b*, *r*; *N*) + *d*(*b*, *x*; *N*) *- d*(*r*, *x*; *N*)]/2.

*(3) Suppose *(*a*, *b*) *is an arc and b is normal. Suppose there is a normal path from a to x *∈ *X which does not include the vertex b. Suppose b has normal paths to y and z in X which are disjoint except at b. Then ω*(*a*, *b*) = [*d*(*r*, *y*; *N*) + *d*(*x*, *z*; *N*) *- d*(*r*, *x*; *N*) *- d*(*y*, *z*; *N*)]/2.

*Proof*. For (1) we take *a *= *x *in Lemma 4.2 and note that *d*(*r*, *y*; *N*) - *d*(*r*, *a*; *N*) = *d*(*a*, *y*; *N*). For (2) we take *b *= *y *= *z *in Lemma 4.2 and note that *d*(*y*, *z*; *N*) = 0. For (3), we use the normal path *a*, *b *as the path from *a *to *b*. □

**Lemma 4.4**. *Assume the hypotheses of Theorem 4.1. Suppose there is a normal path from a to y *∈ *X where a is hybrid with indegree 2 and parents q*_1 _*and q*_2_*. Assume q*_1 _*and q*_2 _*have normal paths to x*_1 _*and x*_2 _*respectively in X. Then d*(*a*, *y*; *N*) = [*d*(*y*, *x*_1_; *N*) + *d*(*y*, *x*_2_; *N*) *- d*(*x*_1_, *x*_2_; *N*)]/2.

*Proof*. See Figure 5b. We first show that the portion of the figure including the paths from *q*_1 _to *x*_1_, from *q*_2 _to *x*_2_, from *a *to *y *and the arcs (*q*_1_, *a*) and (*q*_2_, *a*) accurately represents the hypotheses of the lemma. (The network in Figure 3, which is not normal, has this situation with *a *= 10, *q*_1 _= 5, *q*_2 _= 6, *x*_1 _= *x*_2 _= 4, *y *= 2. Hence Figure 5b is wrong for the network in Figure 3, primarily because the normal paths from *q*_1 _to *x*_1 _and from *q*_2 _to *x*_2 _intersect.) I claim that for normal networks the normal paths from *q*_1 _to *x*_1 _and from *q*_2 _to *x*_2 _have no vertex in common. To see this, suppose there were such a common vertex *w*. In that case by following the normal paths backwards from *w *we infer that either *q*_1 _lies on the path from *q *_2 _to *x*_2 _or else *q*_2 _lies on the path from *q*_1 _to *x*_1_. In the former case there is a directed path from *q*_2 _to *q*_1_, whence the arc (*q*_2_, *a*) is redundant, contradicting the normality of the network. In the latter case (*q*_1_, *a*) is redundant. It follows that the paths are disjoint. In particular, *x*_1 _≠ *x*_2_.

Similarly, neither path can intersect the normal path from *a *to *y*. If, for example, the path from *q*_1 _to *x*_1 _intersected the path from *a *to *y*, then by following the normal paths backwards we would have that either *q*_1 _lies on the path from *a *to *y *or else *a *lies on the path from *q*_1 _to *x*_1_. In the former case there would be a directed cycle from *q*_1 _to *a *to *q*_1_, contradicting that the network is acyclic. In the latter case the hybrid vertex *a *would lie on the normal path from *q*_1 _to *x*_1_, contradicting that it is a normal path.

Suppose *p *∈ *Par*(*N*) is a parent map that satisfies *p*(*a*) = *q*_1_, and let *p*' denote the complementary parent map that agrees with *p *except that *p*'(*a*) = *q*_2_. Thus *N_p _*and *N_p_*_' _agree except that *N_p _*contains the arc (*q*_1_, *a*) while *N_p_*_' _contains instead the arc (*q*_2_, *a*). In particular they both contain the same paths from *q*_1 _to *x*_1_, from *q*_2 _to *x*_2_, and from *a *to *y*. Let *v *= mrca(*q*_1_, *q*_2_; *N_p_*). There is a directed path from *r *to *v *since *r *is the root (possibly *r *= *v*). There are directed paths from *v *to *q*_1 _and *v *to *q*_2 _in *N_p _*which are disjoint except for *v*. Figure 5b thus shows a portion of *N_p _*relevant to the lemma, together with the arc (*q*_2_, *a*).

In *N_p _*we see from Figure 5b that

$$\begin{array}{c} d\left(y,x_{\text{1}};N_{p}\right)=d\left(a,y;N_{p}\right)+w\left(q_{\text{1}},a\right)+d\left(q_{\text{1}},x_{\text{1}};N_{p}\right),\\ d\left(y,x_{\text{2}};N_{p}\right)=d\left(a,y;N_{p}\right)+w\left(q_{\text{1}},a\right)+d\left(q_{\text{1}},q_{\text{2}};N_{p}\right)+d\left(q_{\text{2}},x_{\text{2}};N_{p}\right),\\ d\left(x_{\text{1}},x_{\text{2}};N_{p}\right)=d\left(q_{\text{1}},x_{\text{1}};N_{p}\right)+d\left(q_{\text{1}},q_{\text{2}};N_{p}\right)+d\left(q_{\text{2}},x_{\text{2}};N_{p}\right). \end{array}$$

By substituting these formulas we see that [*d*(*y*, *x*_1_; *N_p_*) + *d*(*y*, *x*_2_; *N_p_*) - *d*(*x*_1_, *x*_2_; *N_p_*)]*/*2 = *d*(*a*, *y*; *N_p_*) + *ω*(*q*_1_, *a*). Since *ω*(*q*_1_, *a*) = 0 because a is hybrid, it follows

$$\left[d\left(y,x_{\text{1}};N_{p}\right)+d\left(y,x_{\text{2}};N_{p}\right)-d\left(x_{\text{1}},x_{\text{2}};N_{p}\right)\right]/\text{2}=d\left(a,y;N_{p}\right)$$

The network *N_p_*_' _is the same except that (*q*_1_, *a*) is replaced by (*q*_2_, *a*). A symmetric argument then shows

$$\begin{array}{c} \left[d\left(y,x_{\text{1}},N_{p^{\prime }}\right)+d\left(y,x_{\text{2}},N_{p^{\prime }}\right)-d\left(x_{\text{1}},x_{\text{2}};N_{p^{\prime }}\right)\right]/)2\\ =d\left(a,y;N_{p^{\prime }}\right)+\omega \left(q_{\text{2}},a\right)=d\left(a,y;N_{p^{\prime }}\right). \end{array}$$

Since the indegree of *a *is 2, every parent map *p *satisfies either *p*(*a*) = *q*_1 _or *p*(*a*) = *q*_2_. It follows that for every *p *∈ *Par*(*N*), [*d*(*y*, *x*_1_; *N_p_*) + *d*(*y*, *x*_2_; *N_p_*) *- d*(*x*_1_, *x*_2_; *N_p_*)]/2 = *d*(*a*, *y*; *N_p_*).

When we take the expected value over all *p *∈ *Par*(*N*) we obtain by linearity [*d*(*y*, *x*_1_; *N*) + *d*(*y*, *x*_2_; *N*) *- d*(*x*_1_, *x*_2_; *N*)]/2 = *d*(*a*, *y*; *N*). □

**Lemma 4.5**. *Assume the hypotheses of Theorem 4.1. Suppose *(*a*, *b*) *is an arc such that b is normal, and a is hybrid with indegree 2 and parents q*_1 _*and q*_2_*. Assume q*_1 _*and q*_2 _*have normal paths to x*_1 _*and x*_2 _*respectively in X. Suppose b has normal paths to w and z in X where the paths are disjoint except for b. Then*

$$\omega \left(a,b\right)=\left[d\left(x_{\text{1}}\text{,}w;N\right)+d\left(x_{\text{2}},z;N\right)-d\left(x_{\text{1}},x_{\text{2}};N\right)-d\left(w,z;N\right)\right]/)2.$$

*Proof*. Since *b *is normal and the paths from *b *to *w *and from *b *to *z *are normal and disjoint except for *b*, we have *d*(*w*, *z*; *N_p_*) = *d*(*b*, *w*; *N_p_*) + *d*(*b*, *z*; *N_p_*) for every parent map *p*, whence *d*(*w*, *z*; *N*) = *d*(*b*, *w*; *N*) + *d*(*b*, *z*; *N*). Similarly *d*(*a*, *w*; *N*) = *ω*(*a*, *b*) + *d*(*b*, *w*; *N*) and *d*(*a*, *z*; *N*) = *ω*(*a*, *b*) + *d*(*b*, *z*; *N*).

Hence [*d*(*a*, *w*; *N*) + *d*(*a*, *z*; *N*) *- d*(*w*, *z*; *N*)]/2

= [*ω*(*a*, *b*) + *d*(*b*, *w*; *N*) + *ω*(*a*, *b*) + *d*(*b*, *z*; *N*) *- d*(*b*, *w*; *N*) *- d*(*b*, *z*; *N*)]/2 = *ω*(*a*, *b*).

In addition, Lemma 4.4 applies with *y *replaced by *w *since the path from *a *to *b *to *w *is normal. Hence *d*(*a*, *w*; *N*) = [*d*(*w*, *x*_1_; *N*) + *d*(*w*, *x*_2_; *N*) *- d*(*x*_1_, *x*_2_; *N*)]/2.

Lemma 4.4 also applies with *y *replaced by *z*. Hence *d*(*a*, *z*; *N*) = [*d*(*z*, *x*_1_; *N*) + *d*(*z*, *x*_2_; *N*) *- d*(*x*_1_, *x*_2_; *N*)]/2.

By substitution it follows *ω*(*a*, *b*) = [*d*(*a*, *w*; *N*) + *d*(*a*, *z*; *N*) *- d*(*w*, *z*; *N*)]/2

= [*d*(*w*, *x*_1_; *N*)+*d*(*w*, *x*_2_; *N*) *- *2*d*(*x*_1_, *x*_2_; *N*)+*d*(*z*, *x*_1_; *N*)+*d*(*z*, *x*_2_; *N*) *- *2*d*(*w*, *z*; *N*)]/4.

But symmetry shows that for each parent map *p*, *d*(*w*, *x*_2_; *N_p_*) + *d*(*z*, *x*_1_; *N_p_*) = *d*(*w*, *x*_1_; *N_p_*) + *d*(*z*, *x*_2_; *N_p_*). Hence by taking the expected value over *p *∈ *Par*(*N*), we have *d*(*w*, *x*_2_; *N*) + *d*(*z*, *x*_1_; *N*) = *d*(*w*, *x*_1_; *N*) + *d*(*z*, *x*_2_; *N*).

Thus *ω*(*a*, *b*) = [2*d*(*w*, *x*_1_; *N*) *- *2*d*(*x*_1_, *x*_2_; *N*) + 2*d*(*z*, *x*_2_; *N*) *- *2*d*(*w*, *z*; *N*)]*/*4 = [*d*(*w*, *x*_1_; *N*) *- d*(*x*_1_, *x*_2_; *N*) + *d*(*z*, *x*_2_; *N*) *- d*(*w*, *z*; *N*)]/2. □

For the next calculations we require a preliminary result. Suppose *h*_0 _is hybrid with indegree 2 and parents *q*_1 _and *q*_2_. For a given parent map *p *with *p*(*h*_0_) = *q*_1_, let *p*' denote the complementary parent map and *G_p _*= *N_p _*∪ *N_p_*_' _be the network *N_p _*with the additional arc (*q*_2_, *h*_0_). Let *H *be the set of hybrid vertices of *N*. For each *p *∈ *Par*(*N*) satisfying *p*(*h*_0_) = *q*_1_, let *W*(*p*) = ∏[*α*(*p*(*h*), *h*): *h *∈ *H*, *h *≠ *h*_0_]. Hence *Pr*(*p*) = *α*(*q*_1_, *h*_0_)*W *(*p*) and *Pr*(*p*') = *α*(*q*_2_, *h*_0_)*W *(*p*).

**Lemma 4.6**. *For any X-network M which is a subnetwork of N, suppose C*(*M*) *is a linear combination of expressions of form d*(*a*, *b*; *M*)*. Then*

*(1) C*(*G_p_*) = *α*(*q*_1_, *h*_0_)*C*(*N_p_*) + *α*(*q*_2_, *h*_0_)*C*(*N_p_*_' _).

*(2) C*(*N*) = ∑[*W*(*p*)*C*(*G_p_*): *p *∈ *Par*(*N*), *p*(*h*_0_) = *q*_1_].

*Proof*. For (1), *d*(*x*, *y*; *G_p_*) = *α*(*q*_1_, *h*_0_)*d*(*x*, *y*; *N_p_*) + *α*(*q*_2_, *h*_0_)*d*(*x*, *y*; *N_p_*_'_). For (2) each term *d*(*a*, *b*; *N*) = *Pr*(*p*)*d*(*a*, *b*; *N_p_*). Hence *C*(*N*) = ∑*Pr*(*p*)*C*(*N_p_*) by linearity

= ∑[*Pr*(*p*)*C*(*N_p_*) + *Pr*(*p*')*C*(*N_p_*_'_): *p*(*h*_0_) = *q*_1_]

= ∑[*α*(*q*_1_, *h*_0_)*W*(*p*)*C*(*N_p_*) + *α*(*q*_2_, *h*_0_)*W*(*p*)*C*(*N_p_*_'_): *p*(*h*_0_) = *q*_1_]

= ∑[*W*(*p*)[*α*(*q*_1_, *h*_0_)*C*(*N_p_*) + *α*(*q*_2_, *h*_0_)*C*(*N_p_*_'_)]: *p*(*h*_0_) = *q*_1_]

= ∑[*W *(*p*)*C*(*G_p_*): *p *∈ *Par*(*N*), *p*(*h*_0_) = *q*_1_]. □

**Lemma 4.7**. *Assume the hypotheses of Theorem 4.1. Suppose a is hybrid with indegree 2 and parents q*_1 _*and q*_2_*. Assume the inheritance is equiprobable at a. Suppose there is a normal path from q*_1 _*to x*_1 _∈ *X, from q*_2 _*to x*_2 _∈ *X, and from a to y *∈ *X. Then d*(*q*_1_, *x*_1_; *N*) = *d*(*x*_1_, *y*; *N*) *- d*(*r*, *y*; *N*)+[*d*(*r*, *x*_1_; *N*)+*d*(*r*, *x*_2_; *N*) *- d*(*x*_1_, *x*_2_; *N*)]/2.

*Proof*. See Figure 5b. As in the proof of Lemma 4.4, the portion of the figure including the paths from *q*_1 _to *x*_1_, from *q*_2 _to *x*_2_, from *a *to *y *and the arcs (*q*_1_, *a*) and (*q*_2_, *a*) accurately represents the hypotheses of the lemma since *N *is normal. Suppose *p *∈ *Par*(*N*) satisfies *p*(*a*) = *q*_1_. Let *p*' denote the complementary parent map such that *p*'(*a*) = *q*_2_. Then all three normal paths in the statement lie in both *N_p _*and *N_p_*_' _since they contain no hybrid arcs. Note that *N_p _*contains (*q*_1_, *a*) and not (*q*_2_, *a*), while *N*_p ' _contains (*q*_2_, *a*) but not (*q*_1_, *a*). Moreover, the path in *N_p _*between *q*_1 _and *q*_2 _must be the same as the path in *N_p_*_' _between *q*_1 _and *q*_2_. Let *v *= mrca(*q*_1_, *q*_2_; *N_p_*); then *v *is also mrca(*q*_1_, *q*_2_; *N_p_*_'_).

For any phylogenetic *X*-network *M *with the same base-set *X *write *L*(*M*) = *d*(*x*_1_, *y*; *M*) *- d*(*r*, *y*; *M*) + [*d*(*r*, *x*_1_; *M*) + *d*(*r*, *x*_2_; *M*) *- d*(*x*_1_, *x*_2_; *M*)]/2.

Note that *L *is a linear expression.

In both *N_p _*and *N_p_*_'_, *d*(*r*, *x*_1_) = *d*(*r*, *v*) + *d*(*v*, *q*_1_) + *d*(*q*_1_, *x*_1_)

*d*(*r*, *x*_2_) = *d*(*r*, *v*) + *d*(*v*, *q*_2_) + *d*(*q*_2_, *x*_2_)

*d*(*x*_1_, *x*_2_) = *d*(*q*_1_, *x*_1_) + *d*(*v*, *q*_1_) + *d*(*v*, *q*_2_) + *d*(*q*_2_, *x*_2_).

Hence [*d*(*r*, *x*_1_) + *d*(*r*, *x*_2_) *- d*(*x*_1_, *x*_2_)]/2 = *d*(*r*, *v*).

In *N_p _*we find *d*(*x*_1_, *y*; *N_p_*) = *d*(*x*_1_, *q*_1_; *N_p_*) + *ω*(*q*_1_, *a*) + *d*(*a*, *y*; *N_p_*), and *d*(*r*, *y*; *N_p_*) = *d*(*r*, *v*; *N_p_*) + *d*(*v*, *q*_1_; *N_p_*) + *ω*(*q*_1_, *a*) + *d*(*a*, *y*; *N_p_*).

Hence *L*(*N_p_*) = *d*(*x*_1_, *y*; *N_p_*) *- d*(*r*, *y*; *N_p_*) + [*d*(*r*, *x*_1_; *N_p_*) + *d*(*r*, *x*_2_; *N_p_*) *- d*(*x*_1_, *x*_2_; *N_p_*)]/2 = *d*(*x*_1_, *y*; *N_p_*) *- d*(*r*, *y*; *N_p_*) + *d*(*r*, *v*; *N_p_*) = *d*(*x*_1_, *q*_1_; *N_p_*) + *ω*(*q*_1_, *a*) + *d*(*a*, *y*; *N_p_*) *- d*(*r*, *v*; *N_p_*) *- d*(*v*, *q*_1_; *N_p_*) *- ω*(*q*_1_, *a*) *- d*(*a*, *y*; *N_p_*) + *d*(*r*, *v*; *N_p_*) = *d*(*x*_1_, *q*_1_; *N_p_*) *- d*(*v*, *q*_1_; *N_p_*).

In *N_p_*_' _we find *d*(*x*_1_, *y*; *N_p_*_'_) = *d*(*q*_1_, *x*_1_; *N_p_*_'_)+*d*(*v*, *q*_1_; *N_p_*_'_)+*d*(*v*, *q*_2_; *N_p_*_'_)+*ω*(*q*_2_, *a*)+*d*(*a*, *y*; *N_p_*_'_) *d*(*r*, *y*; *N_p_*_'_) = *d*(*r*, *v*; *N_p_*_'_) + *d*(*v*, *q*_2_; *N_p_*_'_) + *ω*(*q*_2_, *a*) + *d*(*a*, *y*; *N_p_*_'_).

Hence *L*(*N_p_*_'_) = *d*(*x*_1_, *y*; *N_p_*_'_) *- d*(*r*, *y*; *N_p_*_'_) + [*d*(*r*, *x*_1_; *N_p_*_'_) + *d*(*r*, *x*_2_; *N_p_*_'_) *- d*(*x*_1_, *x*_2_; *N_p_*_'_)]/2 = *d*(*x*_1_, *y*; *N_p_*_'_) *- d*(*r*, *y*; *N_p_*_'_) + *d*(*r*, *v*; *N_p_*_'_) = *d*(*q*_1_, *x*_1_; *N_p_*_'_) + *d*(*v*, *q*_1_; *N_p_*_'_). Thus *L*(*N_p_*) + *L*(*N_p_*_'_) = *d*(*q*_1_, *x*_1_; *N_p_*) *- d*(*v*, *q*_1_; *N_p_*) + *d*(*q*_1_, *x*_1_; *N_p_*_'_) + *d*(*v*, *q*_1_; *N_p_*_'_) = *d*(*q*_1_, *x*_1_; *N_p_*) + *d*(*q*_1_, *x*_1_; *N_p_*_'_) since *d*(*v*, *q*_1_; *N_p_*) = *d*(*v*, *q*_1_; *N_p_*_'_).

Using Lemma 4.6(1) with *h*_0 _= *a*, we see that *L*(*G_p_*) = *α*(*q*_1_, *a*)*L*(*N_p_*) + *α*(*q*_2_, *a*)*L*(*N_p_*_'_) so *L*(*G_p_*) = (1/2)[*L*(*N_p_*) + *L*(*N_p_*_'_)] by equiprobability at *a*.

From above it follows *L*(*G_p_*) = (1/2)*d*(*q*_1_, *x*_1_; *N_p_*) + (1/2)*d*(*q*_1_, *x*_1_; *N_p_*_'_).

By Lemma 4.6(2) *L*(*N*) = ∑[*W*(*p*)*L*(*G *): *p *∈ *Par*(*N*), *p*(*a*) = *q *]

= ∑[*W*(*p*)(1/2)*d*(*q*_1_, *x*_1_; *N_p_*) + *W *(*p*)(1/2)*d*(*q*_1_, *x*_1_; *N_p_*_'_): *p*(*a*) = *q*_1_]

= ∑[*Pr*(*p*)*d*(*q*_1_, *x*_1_; *N_p_*) + *Pr*(*p*')*d*(*q*_1_, *x*_1_; *N_p_*_'_): *p *∈ *Par*(*N*), *p*(*a*) = *q*_1_]

= ∑[*Pr*(*p*)*d*(*q*_1_, *x*_1_; *N_p_*): *p *∈ *Par*(*N*)]

= *d*(*q*_1_, *x*_1_; *N*). □

It is interesting in the proof that different choices of the parent map *p *may yield different vertices *v*; nevertheless all these choices cancel out.

**Lemma 4.8**. *Assume the hypotheses of Theorem 4.1. Suppose h is hybrid with indegree 2 and parents q*_1 _*and q*_2_*. Assume equiprobable inheritance at h. Suppose there is a normal path from q*_2 _*to x*_2 _∈ *X and from h to y *∈ *X. Suppose q*_1 _*has normal child b and there are normal paths from b to z*_1 _∈ *X and from b to z*_2 _∈ *X such that these paths intersect only at b. Then ω*(*q*_1_, *b*) = [2*d*(*z*_1_, *y*; *N*) *- *4*d*(*r*, *y*; *N*) + *d*(*r*, *z*_1_; *N*) + 2*d*(*r*, *x*_2_; *N*) *- d*(*z*_1_, *x*_2_; *N*) + 2*d*(*z*_2_, *y*; *N*) + *d*(*r*, *z*_2_; *N*) *- d*(*z*_2_, *x*_2_; *N*) *- *2*d*(*z*_1_, *z*_2_; *N*)]/4.

*In particular, if b is a leaf, then ω*(*q*_1_, *b*) = [2*d*(*b*, *y*; *N*) *- *2*d*(*r*, *y*; *N*) + *d*(*r*, *b*; *N*) + *d*(*r*, *x*_2_; *N*) *- d*(*b*, *x*_2_; *N*)]/2.

*Proof*. By an argument like that for Lemma 4.2, for each *p *∈ *Par*(*N*) we have *ω*(*q*_1_, *b*) = [*d*(*q*_1_, *z*_1_; *N_p_*) + *d*(*q*_1_, *z*_2_; *N_p_*) *- d*(*z*_1_, *z*_2_; *N_p_*)]/2

whence by averaging over *p *∈ *Par*(*N*) we find *ω*(*q*_1_, *b*) = [*d*(*q*_1_, *z*_1_; *N*) + *d*(*q*_1_, *z*_2_; *N*) *- d*(*z*_1_, *z*_2_; *N*)]/2.

But the paths from *q*_1 _to *z*_1 _and from *q*_2 _to *z*_2 _are normal, so by Lemma 4.7 *d*(*q*_1_, *z*_1_; *N*) = *d*(*z*_1_, *y*; *N*) *-d*(*r*, *y*; *N*)+[*d*(*r*, *z*_1_; *N*)+ *d*(*r*, *x*_2_; *N*) *-d*(*z*_1_, *x*_2_; *N*)]/2 and *d*(*q*_1_, *z*_2_; *N*) = *d*(*z*_2_, *y*; *N*)*-d*(*r*, *y*; *N*)+[*d*(*r*, *z*_2_; *N*)+*d*(*r*, *x*_2_; *N*)*-d*(*z*_2_, *x*_2_; *N*)]/2. Hence *ω*(*q*_1_, *b*) = [*d*(*z*_1_, *y*; *N*)*-d*(*r*, *y*; *N*)+[*d*(*r*, *z*_1_; *N*)+*d*(*r*, *x*_2_; *N*)*-d*(*z*_1_, *x*_2_; *N*)]/2 +*d*(*z*_2_, *y*; *N*)*-d*(*r*, *y*; *N*)+[*d*(*r*, *z*_2_; *N*)+*d*(*r*, *x*_2_; *N*)*-d*(*z*_2_, *x*_2_; *N*)]/2*-d*(*z*_1_, *z*_2_; *N*)]/2 = [2*d*(*z*_1_, *y*; *N*)*-*2*d*(*r*, *y*; *N*)+*d*(*r*, *z*_1_; *N*)+*d*(*r*, *x*_2_; *N*)*-d*(*z*_1_, *x*_2_; *N*)+2*d*(*z*_2_, *y*; *N*)*-*2*d*(*r*, *y*; *N*) + *d*(*r*, *z*_2_; *N*) + *d*(*r*, *x*_2_; *N*) *- d*(*z*_2_, *x*_2_; *N*) *- *2*d*(*z*_1_, *z*_2_; *N*)]/4 = [2*d*(*z*_1_, *y*; *N*)*-*4*d*(*r*, *y*; *N*)+*d*(*r*, *z*_1_; *N*)+2*d*(*r*, *x*_2_; *N*)*-d*(*z*_1_, *x*_2_; *N*)+2*d*(*z*_2_, *y*; *N*)+ *d*(*r*, *z*_2_; *N*) *- d*(*z*_2_, *x*_2_; *N*) *- *2*d*(*z*_1_, *z*_2_; *N*)]/4.

If *b *is a leaf we may take *b *= *z*_1 _= *z*_2 _to obtain *ω*(*q*_1_, *b*) = [2*d*(*b*, *y*; *N*) *- *4*d*(*r*, *y*; *N*) + *d*(*r*, *b*; *N*) + 2*d*(*r*, *x*_2_; *N*) *- d*(*b*, *x*_2_; *N*) + 2*d*(*b*, *y*; *N*) + *d*(*r*, *b*; *N*) *- d*(*b*, *x*_2_; *N*) *- *2*d*(*b*, *b*; *N*)]/4 = [4*d*(*b*, *y*; *N*)*-*4*d*(*r*, *y*; *N*)+2*d*(*r*, *b*; *N*)+2*d*(*r*, *x*_2_; *N*)*-*2*d*(*b*, *x*_2_; *N*)*-*2*d*(*b*, *b*; *N*)]/4 = [2*d*(*b*, *y*; *N*) *- *2*d*(*r*, *y*; *N*) + *d*(*r*, *b*; *N*) + *d*(*r*, *x*_2_; *N*) *- d*(*b*, *x*_2_; *N*)]/2. □

We next prove analogues of Lemma 4.7 and Lemma 4.8 for the case where the hybrid is not equiprobable and we are dealing with the situation in Figure 2 rather than Figure 5b.

**Lemma 4.9**. *Assume the hypotheses of Theorem 4.1. Suppose h*_0 _*is hybrid with indegree 2 and parents q*_1 _*and q*_2_*. Suppose there is a normal path from q*_1 _*to x*_1 _∈ *X, from q*_2 _*to x*_2 _∈ *X, and from h to y *∈ *X. Assume q*_3 _*is such that there is a normal path from q*_3 _*to q*_2_*, a normal path from q*_3 _*to x*_3 _∈ *X, but no directed path from q*_3 _*to q*_1_*. Suppose M is a phylogenetic X-network that is a subnetwork of N. Let*

*(a) w_rv_*(*M*) = [*d*(*r*, *x*_1_; *M*) + *d*(*r*, *x*_3_; *M*) *- d*(*x*_1_, *x*_3_; *M*)]/2 = [*d*(*r*, *x*_1_; *M*) + *d*(*r*, *x*_2_; *M*) *- d*(*x*_1_, *x*_2_; *M*)]/2

*(b) *$w_{vq_{3}}\left(M\right)=\left[d\left(r,x_{\text{3}};M\right)+d\left(x_{\text{1}},x_{\text{2}};M\right)-d\left(r,x_{\text{1}};M\right)-d\left(x_{\text{3}},x_{\text{2}};M\right)\right]/\text{2}$

*(c) *$w_{q_{3}x_{3}}\left(M\right)=\left[d\left(r,x_{\text{3}};M\right)+d\left(x_{\text{3}},x_{\text{2}};M\right)-d\left(r,x_{\text{2}};M\right)\right]/\text{2}$

*(d) w_hy_*(*M*) = [*d*(*y*, *x*_2_; *M*) + *d*(*y*, *x*_1_; *M*) *- d*(*x*_1_, *x*_2_; *M*)]/2

*(e) E*_2_(*M*) = *d*(*x*_1_, *y*; *M*) *- d*(*r*, *y*; *M*) + *w_rv_*(*M*)

*(f) E*_4_(*M*) = *d*(*x*_2_, *y*; *M*) *- d*(*r*, *y*; *M*) + *w_rv_*(*M*)

*(g) *$\alpha \left(M\right)=\left[\text{2}d\left(x_{\text{3}},y;M\right)-\text{2}w_{q_{\text{3}}x_{\text{3}}}\left(M\right)-\text{2}w_{hy}\left(M\right)-d\left(r,x_{\text{1}};M\right)+E_{\text{2}}\left(M\right)+\right)$

$\left(\text{2}w_{rv}\left(M\right)+E_{\text{4}}\left(M\right)-d\left(r,x_{\text{2}};M\right)+\text{ 2}w_{vq_{3}}\left(M\right)\right]/)\left[\text{4}w_{vq_{3}}\left(M\right)\right]$

*(h) *$w_{vq_{1}}\left(M\right)=\left[d\left(r,x_{\text{1}};M\right)-E_{\text{2}}\left(M\right)-w_{rv}\left(M\right)\right]/)\left[\text{2}a\left(M\right)\right]$

*(i) *$w_{q_{\text{3}}q_{2}}\left(M\right)=\left[d\left(x_{\text{3}},y;M\right)-w_{q_{\text{3}}x_{\text{3}}}\left(M\right)-w_{hy}\left(M\right)-a\left(M\right)\left(w_{vq_{3}}\left(M\right)+w_{vq_{1}}\left(M\right)\right)\right]/)\left(\text{1}-a\left(M\right)\right)$

*(j) *$w_{q_{\text{1}}x_{1}}\left(M\right)=d\left(r,x_{\text{1}};M\right)-w_{rv}\left(M\right)-w_{vq_{1}}\left(M\right)$

*(k) *$w_{q_{\text{2}}x_{2}}\left(M\right)=d\left(r,x_{\text{2}};M\right)-w_{rv}\left(M\right)-w_{vq_{3}}\left(M\right)-w_{q_{\text{3}}q_{2}}\left(M\right)$

*(l) *$C\left(M\right)=\text{2}d\left(x_{\text{3}},y;M\right)-\text{2}w_{q_{3}x_{3}}\left(M\right)-\text{2}w_{hy}\left(M\right)-d\left(r,x_{\text{1}};M\right)+E_{\text{2}}\left(M\right)+$

$\text{2}w_{rv}\left(M\right)\;+E_{\text{4}}\left(M\right)-d\left(r,x_{\text{2}};M\right)\;+\text{ 2}w_{vq_{3}}\left(M\right)$

*(m) *$D\left(M\right)=\text{4}w_{vq_{3}}\left(M\right)$.

Then

*(i) α*(*q*_1_, *h*; *N*) = *α*(*N*) = *C*(*N*) */D*(*N*).

*(ii) *$d\left(q_{\text{1}},x_{\text{1}};N\right)=w_{q_{1}x_{1}}\left(N\right)$.

*(iii) *$d\left(q_{\text{2}},x_{\text{2}};N\right)=w_{q_{2}x_{2}}\left(N\right)$.

*Proof*. Suppose *p *∈ *Par*(*N*) is a parent map satisfying *p*(*h*_0_) = *q*_1 _and *p*' is the complementary parent map agreeing with *p *except that *p*' (*h*_0_) = *q*_2_. Let *G_p _*= *N_p _*with the additional arc (*q*_2_, *h*_0_), so *G_p _*= *N_p _*∪ *N_p_*_'_. A portion of *G_p _*is shown in Figure 2. Note that Figure 2 is accurate for every *p *(although the vertex *v *may differ for different *p*) because of the hypotheses on *q*_1_, *q*_2_, *q*_3_, *h*_0_, *x*_1_, *x*_2_, *x*_3_, and *y*.

Write *u_rv _*= *d*(*r*, *v*; *G_p_*), $u_{vq_{1}}=d\left(v,q_{\text{1}};G_{p}\right)$, $u_{vq_{3}}=d\left(v,q_{\text{3}};G_{p}\right)$, $u_{q_{3}x_{3}}=d\left(q_{\text{3}},x_{\text{3}};G_{p}\right)$, $u_{q_{3}q_{2}}=d\left(q_{\text{3}},q_{\text{2}};G_{p}\right)$, $u_{q_{2}x_{2}}=d\left(q_{\text{2}},x_{\text{2}};G_{p}\right)$, *u_hy _*= *d*(*h*, *y*; *G_p_*), $u_{q_{1}x_{1}}=d\left(q_{\text{1}},x_{\text{1}};G_{p}\right)$.

The definition of the tree-average distance yields the following ten equations for *G_p_*, where *α *= *α*(*q*_1_, *h*_0_).

$d\left(r,x_{\text{1}};G_{p}\right)=u_{rv}+u_{vq_{\text{1}}}+u_{q_{\text{1}}x_{\text{1}}}$

$d\left(r,x_{\text{3}};G_{p}\right)\;=u_{rv}+u_{vq_{\text{3}}}+u_{q_{\text{3}}x_{\text{3}}}$

$d\left(r,x_{\text{2}};G_{p}\right)=u_{rv}+u_{vq_{\text{3}}}+u_{q_{\text{3}}q_{\text{2}}}+u_{q_{\text{2}}x_{\text{2}}}$

$\begin{array}{l} d\left(r,y;G_{p}\right)=\alpha \left[u_{rv}+u_{vq_{1}}+u_{hy}\right]+\left(\text{1}-\alpha \right)\left[u_{rv}+u_{vq_{3}}+u_{q_{\text{3}}q_{2}}+u_{hy}\right]\\ =u_{rv}+u_{hy}+\alpha_{uvq_{\text{1}}}+\left(\text{1}-\alpha \right)\left(u_{vq_{\text{3}}}+u_{q_{\text{3}}q_{\text{2}}}\right) \end{array}$

$d\left(x_{\text{1}},x_{\text{3}};G_{p}\right)=u_{q_{\text{1}}x_{\text{1}}}+u_{vq_{\text{1}}}+u_{vq_{\text{3}}}+u_{q_{\text{3}}x_{\text{3}}}$

$d\left(x_{\text{1}},x_{\text{2}};G_{p}\right)=u_{q_{\text{1}}x_{\text{1}}}+u_{vq_{\text{1}}}+u_{vq_{\text{3}}}+u_{q_{\text{3}}q_{\text{2}}}+u_{q_{\text{2}}x_{\text{2}}}$

$\begin{array}{l} d\left(x_{\text{1}},y;G_{p}\right)=\alpha \left[u_{q_{\text{1}}x_{\text{1}}}+u_{hy}\right]+\left(\text{1}-\alpha \right)\left[u_{q_{\text{1}}x_{\text{1}}}+u_{vq_{\text{1}}}+u_{vq_{\text{3}}}+u_{q_{\text{3}}q_{\text{2}}}+u_{hy}\right]\\ =u_{q_{\text{1}}x_{\text{1}}}+u_{hy}+\left(\text{1}-\alpha \right)\left[u_{vq_{\text{1}}}+u_{vq_{\text{3}}}+u_{q_{\text{3}}q_{\text{2}}}\right] \end{array}$

$d\left(x_{\text{3}},x_{\text{2}};G_{p}\right)=u_{q_{\text{3}}x_{\text{3}}}+u_{q_{\text{3}}q_{\text{2}}}+u_{q_{\text{2}}x_{\text{2}}}$

$\begin{array}{l} d\left(x_{\text{3}},y;G_{p}\right)=\alpha \left[u_{q_{\text{3}}x_{\text{3}}}+u_{vq_{\text{3}}}+u_{vq_{\text{1}}}+u_{hy}\right]+\left(\text{1}-\alpha \right)\left[u_{q_{\text{3}}x_{\text{3}}}+u_{q_{\text{3}}q_{\text{2}}}+u_{hy}\right]\\ =u_{q_{\text{3}}x_{\text{3}}}+u_{hy}+\alpha \left(u_{vq_{\text{3}}}+u_{vq_{\text{1}}}\right)+\left(\text{1}-\alpha \right)\left(u_{q_{\text{3}}q_{\text{2}}}\right) \end{array}$

$\begin{array}{l} d\left(x_{\text{2}},y;G_{p}\right)=\alpha \left[u_{q_{\text{2}}x_{\text{2}}}+u_{q_{\text{3}}q_{\text{2}}}+u_{vq_{\text{3}}}+u_{vq_{\text{1}}}+u_{hy}\right]+\left(\text{1}-\alpha \right)\left[u_{q_{\text{2}}x_{\text{2}}}+u_{hy}\right]\\ =u_{q_{\text{2}}x_{\text{2}}}+u_{hy}+\alpha \left(u_{q_{\text{3}}q_{\text{2}}}+u_{vq_{\text{3}}}+u_{vq_{\text{1}}}\right) \end{array}$

We now solve this system of ten equations.

It is straightforward by simplifying the expressions that [*d*(*r*, *x*_1_; *G_p_*) + *d*(*r*, *x*_3_; *G_p_*) *- d*(*x*_1_, *x*_3_; *G_p_*)]/2 = *u_rv _*so a comparison with (a) shows that *w_rv_*(*G_p_*) = *u_rv_*. Similarly [*d*(*r*, *x*_1_; *G_p_*) + *d*(*r*, *x*_2_; *G_p_*) *- d*(*x*_1_, *x*_2_; *G_p_*)]/2 = *u_rv _*so the two expressions in (a) for *w_rv_*(*G_p_*) are the same.

Likewise from the ten equations, $\left[d\left(r,x_{\text{3}};G_{p}\right)+d\left(x_{\text{1}},x_{\text{2}};G_{p}\right)-d\left(r,x_{\text{1}};G_{p}\right)-d\left(x_{\text{3}},x_{\text{2}};G_{p}\right)\right]/\text{2}=u_{vq_{3}}$

so $w_{vq_{\text{3}}}\left(G_{p}\right)=u_{vq_{\text{3}}}$;

$\left[d\left(r,x_{\text{3}};G_{p}\right)+d\left(x_{\text{3}},x_{\text{2}};G_{p}\right)-d\left(r,x_{\text{2}};G_{p}\right)\right]/\text{2}=u_{q_{3}x_{3}}$

so $w_{q_{\text{3}}x_{\text{3}}}\left(G_{p}\right)=u_{q_{\text{3}}x_{\text{3}}}$;

[*d*(*y*, *x*_2_; *G_p_*) + *d*(*y*, *x*_1_; *G_p_*) *- d*(*x*_1_, *x*_2_; *G_p_*)]/2 = *u_hy _*so *w_hy_*(*G_p_*) = *u_hy_*.

From the system of ten equations we see

$E_{\text{2}}\left(G_{p}\right)=u_{q_{\text{1}}x_{\text{1}}}+\left(\text{1}-\alpha \right)u_{vq_{\text{1}}}-\alpha_{uvq_{\text{1}}}=u_{q_{\text{1}}x_{\text{1}}}+\left(\text{1}-\text{2}\alpha \right)u_{vq_{\text{1}}}.$

Since $d\left(r,x_{\text{1}};G_{p}\right)=u_{rv}+u_{vq_{1}}+u_{q_{\text{1}}x_{1}}$ it follows $d\left(r,x_{\text{1}};G_{p}\right)=u_{rv}+u_{vq_{1}}+E_{\text{2}}\left(G_{p}\right)-\left(\text{1}-\text{2}\alpha \right)u_{vq_{1}}$ whence

(1) $$\text{2}\alpha u_{vq_{1}}=d\left(r,x_{\text{1}};G_{p}\right)-E_{\text{2}}\left(G_{p}\right)-u_{rv}$$

Similarly $E_{\text{4}}\left(G_{p}\right)=u_{q_{\text{2}}x_{\text{2}}}+u_{hy}+\alpha \left(u_{q_{\text{3}}q_{\text{2}}}+u_{vq_{\text{3}}}+u_{vq_{\text{1}}}\right)-u_{rv}-u_{hy}-\alpha u_{vq_{\text{1}}}-\left(\text{1}-\alpha \right)\left(u_{vq_{\text{3}}}+u_{q_{\text{3}}q_{\text{2}}}\right)+u_{rv}$

$=u_{q_{\text{2}}x_{\text{2}}}+\alpha \left(u_{q_{\text{3}}q_{\text{2}}}+u_{vq_{\text{3}}}\right)-\left(\text{1}-\alpha \right)\left(u_{vq_{\text{3}}}+u_{q_{\text{3}}q_{\text{2}}}\right)=u_{q_{\text{2}}x_{\text{2}}}+\left(\text{2}\alpha -\text{1}\right)\left(u_{vq_{\text{3}}}+u_{q_{\text{3}}q_{\text{2}}}\right)$.

But from $d\left(r,x_{\text{2}};G_{p}\right)\;=u_{rv}+u_{vq_{3}}+u_{q_{\text{3}}q_{2}}+u_{q_{2}x_{2}}$ it follows $u_{q_{2}x_{2}}=d\left(r,x_{\text{2}};G_{p}\right)-u_{rv}-u_{vq_{3}}-u_{q_{3}q_{2}}$ so $E_{\text{4}}\left(G_{p}\right)=d\left(r,x_{\text{2}};G_{p}\right)-u_{rv}-u_{vq_{3}}-u_{q_{3}q_{2}}+\left(\text{2}\alpha -\text{1}\right)\left(u_{vq_{3}}+u_{q_{3}q_{2}}\right)$. This can be solved to show

(2) $$\left(\text{2}-\text{2}\alpha \right)\left(u_{vq_{3}}+u_{q_{3}q_{2}}\right)=d\left(r,x_{\text{2}};G_{p}\right)-u_{rv}-E_{\text{4}}\left(G_{p}\right)$$

Since $d\left(x_{\text{3}},y;G_{p}\right)=u_{q_{3}x_{3}}+u_{hy}+a\left(u_{vq_{3}}+u_{vq_{1}}\right)+\left(\text{1}-\alpha \right)\left(u_{q_{3}q_{2}}\right)$ we obtain

(3) $$\alpha \left(u_{vq_{3}}+u_{vq_{1}}\right)+\left(\text{1}-\alpha \right)\left(u_{q_{3}q_{2}}\right)=d\left(x_{\text{3}},y\right)-u_{q_{3}x_{3}}-u_{hy}$$

Note (1), (2), and (3) are equations in the unknowns *α*, $w_{vq_{1}}$,$w_{q_{3}q_{2}}$ in terms of known quantities such as *w_rv_*, $w_{q_{3}x_{3}}$, *w_hy_*, $w_{vq_{3}}$, *E*_4_(*G_p_*). These three equations in three unknowns can be solved to yield for *G_p _*(for any *p *∈ *Par*(*N*) with *p*(*h*) = *q*_1_) the following:

$\begin{array}{c} \alpha \left(G_{p}\right)=[\text{2}d\left(x_{\text{3}},y;G_{p}\right)-\text{2}w_{q_{3}x_{3}}-\text{2}w_{hy}-d\left(r,x_{\text{1}};G_{p}\right)+E_{\text{2}}\left(G_{p}\right)+\text{2}w_{rv}+E_{\text{4}}\left(G_{p}\right)-\\ d\left(r,x_{\text{2}};G_{p}\right)+\text{2}w_{vq_{3}}]/\left[\text{4}w_{vq_{3}}\right] \end{array}$

$w_{vq_{1}}\left(G_{p}\right)=\left[d\left(r,x_{\text{1}};G_{p}\right)-E_{\text{2}}\left(G_{p}\right)-w_{rv}\right]/\left[\text{2}\alpha \left(G_{p}\right)\right]$

$w_{q_{3}q_{2}}\left(G_{p}\right)=\left[d\left(x_{\text{3}},y;G_{p}\right)-w_{q_{3}x_{3}}-w_{hy}-\alpha \left(w_{vq_{3}}+w_{vq_{1}}\right)\right]/)\left(\text{1}-\alpha \left(G_{p}\right)\right)$.

Moreover, the value of *α *is independent of the choice of *p*.

We thus have *C*(*G_p_*) = *αD*(*G_p_*) for each *p *satisfying *p*(*h*_0_) = *q*_1_.

By Lemma 4.6, *C*(*N*) = ∑[*W*(*p*)*C*(*G_p_*): *p*(*h*_0_) = *q*_1_] and *D*(*N*) = ∑[*W*(*p*)*D*(*G_p_*): *p*(*h*_0_) = *q*_1_].

Hence *C*(*N*) = ∑[*W*(*p*) *αD*(*G_p_*): *p*(*h*_0_) = *q*_1_] = *α *∑[*W*(*p*)*D*(*G_p_*): *p*(*h*_0_) = *q*_1_] = *αD*(*N*).

It follows that *α *= *C*(*N*) ≠ *D*(*N*). This proves (i).

Similarly, for any *p *∈ *Par*(*N*) satisfying *p*(*h*_0_) = *q*_1_, since the path from *q*_1 _to *x*_1 _is normal, $d\left(q_{\text{1}},x_{\text{1}};N\right)=d\left(q_{\text{1}},x_{\text{1}};G_{p}\right)=w_{q_{1}x_{1}}\left(G_{p}\right)$. By Lemma 4.6 *d*(*q*_1_, *x*_1_; *N*) = ∑[*W*(*p*)*d*(*q*_1_, *x*_1_; *G_p_*): *p *∈ *Par*(*N*), *p*(*h*_0_) = *q*_1_] $=\sum \left[W\left(p\right)w_{q_{1}x_{1}}\left(G_{p}\right):p\in Par\left(N\right),p\left(h_{0}\right)=q_{\text{1}}\right]=w_{q_{1}x_{1}}\left(N\right)$, proving (ii). Similarly $d\left(q_{\text{2}},x_{\text{2}};N\right)=w_{q_{2}x_{2}}\left(N\right)$, proving (iii). □

**Lemma 4.10**. *Assume the hypotheses of Theorem 4.1. Suppose h*_0 _*is hybrid with indegree 2 and parents q*_1 _*and q*_2_*. Suppose there is a normal path from q*_3 _*to q*_2_*, from q*_2 _*to x*_2 _∈ *X, from q*_1 _*to x*_1 _∈ *X, from h*_0 _*to y *∈ *X, and from q*_3 _*to x*_3 _∈ *X but no directed path from q*_3 _*to q*_1_ .

*(a) Suppose q*_1 _*has normal child b and there are normal paths from b to x*_1 _∈ *X and from b to z*_1 _∈ *X such that these paths intersect only at b. Then ω*(*q*_1_, *b*) = [*d*(*q*_1_, *x*_1_; *N*) + *d*(*q*_1_, *z*_1_; *N*) *- d*(*x*_1_, *z*_1_; *N*)]/2*, where d*(*q*_1_, *x*_1_; *N*) *and d*(*q*_1_, *z*_1_; *N*) *are determined by Lemma 4.9*.

*(b) Suppose q*_2 _*has normal child c and there are normal paths form c to x*_2 _∈ *X and from c to z*_2 _∈ *X such that these paths intersect only at c. Then ω*(*q*_2_, *c*) = [*d*(*q*_2_, *x*_2_; *N*) + *d*(*q*_2_, *z*_2_; *N*) *- d*(*x*_2_, *z*_2_; *N*)]/2*, where d*(*q*_2_, *x*_2_; *N*) *and d*(*q*_2_, *z*_2_; *N*) *are determined by Lemma 4.9*.

*Proof*. For (a), Lemma 4.9 applies to yield *d*(*q*_1_, *x*_1_; *N*). By a parallel computation with *z*_1 _replacing *x*_1_, Lemma 4.9 also yields *d*(*q*_1_, *z*_1_; *N*). Since the paths from *q*_1 _to *x*_1 _and *z*_1 _are normal, it follows that *ω*(*q*_1_, *b*) = *d*(*q*_1_, *b*; *N*) = [*d*(*q*_1_, *x*_1_; *N*)+*d*(*q*_1_, *z*_1_; *N*)*-d*(*x*_1_, *z*_1_; *N*)]/2 by an argument like that of Lemma 4.2. A similar argument shows (b). □

We now turn to the proof of the main theorem 4.1:

*Proof*. We seek to reconstruct each weight *ω *(*a*, *b*) and each probability. If *b *is hybrid, then by assumption *ω *(*a*, *b*) = 0. Hence we may assume *b *is normal.

At the tail *a *we have the following exhaustive list of possibilities:

Case *A*_1_. There is a normal path from *a *to some *w *∈ *X *such that the path does not go through *b*. This includes the possibility where *a *∈ *X *(in which case the trivial path at *a *satisfies the condition). Since *r *∈ *X*, this includes the case *a *= *r*.

Case *A*_2_. *a *is hybrid and *b *is its unique child. Since *a *is hybrid it has two parents *q*_1 _and *q*_2_. Choose a normal path from *q*_1 _to *w*_1 _∈ *X *and from *q*_2 _to *w*_2 _∈ *X*.

Case *A*_3_. *a *has a hybrid child *h*' with other parent *q*'. Choose a normal path from *q*' to *w*_1 _∈ *X *and from *h*' to *w*_2 _∈ *X*.

At the head *b*, either *b *∈ *X *or else *b *is not a leaf and *b *has at least two children, at least one of which must be normal. Hence we have the following exhaustive list of possibilities:

Case *B*_1_. *b *∈ *X*.

Case *B*_2_. *b *has two normal children *c*_1 _and *c*_2_. For *i *= 1, 2 there is a normal path from *c_i _*to *x_i _*∈ *X*.

Case *B*_3_. *b *has one normal child *c *and a hybrid child *h *for which there is exactly one other parent *q*. There is a normal path from *c *to *x *∈ *X*, from *h *to *y *∈ *X*, and from *q *to *z *∈ *X*.

Since there are 3 cases for *a *and three cases for *b*, we must consider 9 cases. The case where *A_i _*is combined with *B_j _*will be denoted Case *A_i_B_j_*. We will compute *ω*(*a*, *b*). To compute the probabilities, it suffices to compute *α*(*a*, *h*') in situation *A*_3_.

Case *A*_1_*B*_1_. Assume there is a normal path from *a *to some *w *∈ *X *such that the path does not go through *b*, and *b *∈ *X*. Then Lemma 4.3(2) shows that *ω*(*a*, *b*) = [*d*(*r*, *b*; *N*) + *d*(*w*, *b*; *N*) *- d*(*r*, *w*; *N*)]/2.

Case *A*_1_*B*_2_. Assume there is a normal path from *a *to some *w *∈ *X *such that the path does not go through *b*. Assume *b *has two normal children *c*_1 _and *c*_2_. For *i *= 1, 2 there is a normal path from *c_i _*to *x_i _*∈ *X*. In this case, Lemma 4.3(3) shows that *ω*(*a*, *b*) = [*d*(*r*, *x*_1_; *N*) + *d*(*w*, *x*_2_; *N*) *- d*(*r*, *w*; *N*) *- d*(*x*_1_, *x*_2_; *N*)]/2.

Case *A*_2_*B*_1_. Assume *a *is hybrid and *b *is its unique child. Assume *b *∈ *X*. Since *a *is hybrid it has two parents *q*_1 _and *q*_2_. Choose a normal path from *q*_1 _to *w*_1 _∈ *X *and from *q*_2 _to *w*_2 _∈ *X*. In this case, Lemma 4.4 shows that *ω*(*a*, *b*) = [*d*(*b*, *w*_1_; *N*) + *d*(*b*, *w*_2_; *N*) *- d*(*w*_1_, *w*_2_; *N*)]/2.

Case *A*_2_*B*_2_. Assume *a *is hybrid and *b *is its unique child. Since *a *is hybrid it has two parents *q*_1 _and *q*_2_. Choose a normal path from *q*_1 _to *w*_1 _∈ *X *and from *q*_2 _to *w*_2 _∈ *X*. Assume *b *has two normal children *c*_1 _and *c*_2_. For *i *= 1, 2 there is a normal path from *c_i _*to *x_i _*∈ *X*. In this case by Lemma 4.5 we have *ω*(*a*, *b*) = [*d*(*w*_1_, *x*_1_; *N*) + *d*(*w*_2_, *x*_2_; *N*) *- d*(*w*_1_, *w*_2_; *N*) *- d*(*x*_1_, *x*_2_; *N*)]/2.

Case *A*_3_*B*_1_. Assume *a *has a hybrid child *h*' with other parent *q*'. Choose a normal path from *q*' to *w*_1 _∈ *X *and from *h*' to *w*_2 _∈ *X*. Assume *b *∈ *X*. In the equiprobable case, Lemma 4.7 with *q*_1 _= *a*, *x*_1 _= *b*, *x*_2 _= *w*_2 _shows *ω*(*a*, *b*) = *d*(*a*, *b*; *N*) = *d*(*b*, *w*_2_; *N*) *- d*(*r*, *w*_2_; *N*) + [*d*(*r*, *b*; *N*) + *d*(*r*, *w*_1_; *N*) *- d*(*b*, *w*_1_; *N*)]/2.

In the other case, Lemma 4.9(ii) with *q*_1 _= *a *and *x*_1 _= *b *yields *ω*(*a*, *b*) while Lemma 4.9(i) yields *α*(*a*, *h*').

Case *A*_3_*B*_2_. Assume *a *has a hybrid child *h*' with other parent *q*'. Choose a normal path from *q*' to *w*_1 _∈ *X *and from *h*' to *w*_2 _∈ *X*. Assume *b *has two normal children *c*_1 _and *c*_2_. For *i *= 1, 2 there is a normal path from *c_i _*to *x_i _*∈ *X*. In the equiprobable case, Lemma 4.8 with *q*_1 _= *a*, *y *= *w*_2_, *z*_1 _= *x*_1_, *z*_2 _= *x*_2_, *h *= *h*', *q*_2 _= *q*', *x*_2 _= *w*_1 _shows *ω*(*a*, *b*) = [2*d*(*x*_1_, *w*_2_; *N*) *- *4*d*(*r*, *w*_2_; *N*)+*d*(*r*, *x*_1_; *N*)+2*d*(*r*, *w*_1_; *N*) *- d*(*x*_1_, *w*_1_; *N*)+ 2*d*(*x*_2_, *w*_2_; *N*) + *d*(*r*, *x*_2_; *N*) *- d*(*x*_2_, *w*_1_) *- *2*d*(*x*_1_, *x*_2_)]*/*4.

In the non-equiprobable case Lemma 4.10a applies to determine *ω*(*a*, *b*), while Lemma 4.9(i) determines *α*(*a*, *h*').

**Figure 6:**
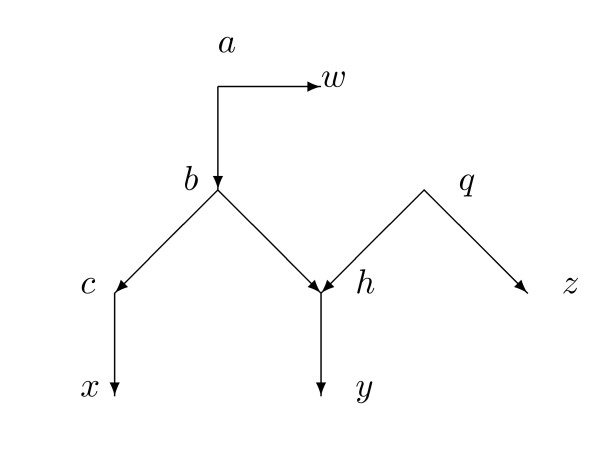
**Case *A*_1_*B*_3_**.

Case *A*_1_*B*_3_. Assume that there is a normal path from *a *to some *w *∈ *X *such that the path does not go through *b*. Assume *b *has one normal child *c *and a hybrid child *h *for which there is exactly one other parent *q*. There is a normal path from *c *to *x *∈ *X*, from *h *to *y *∈ *X*, and from *q *to *z *∈ *X*. See Figure 6. Since *N *is normal, an argument like that for Lemma 4.4 shows that Figure 6 is accurate for the situation.

In this situation, by Lemma 4.4(2), *d*(*a*, *x*; *N*) = [*d*(*x*, *r*; *N*) + *d*(*x*, *w*; *N*) *- d*(*r*, *w*; *N*)]/2. In the equiprobable case, by Lemma 4.7, with *b *= *q*_1_, *x*_1 _= *x*, *z *= *x*_2_, *d*(*b*, *x*; *N*) = *d*(*x*, *y*; *N*) *- d*(*r*, *y*; *N*) + [*d*(*r*, *x*; *N*) + *d*(*r*, *z*; *N*) *- d*(*x*, *z*; *N*)]/2.

Finally *ω*(*a*, *b*) = *d*(*a*, *x*; *N*) *- d*(*b*, *x*; *N*). In the non-equiprobable case, Lemma 4.9 with *a *= *q*_3 _and *b *= *q*_2 _yields the computation of $w\left(a,b\right)=w_{q_{3},q_{2}}\left(N\right)$ and Lemma 4.9(i) shows *α*(*b*, *h*) = *α*(*q*_2_, *h*; *N*) = 1 *- α*(*q*_1_, *h*; *N*).

Case *A*_2_*B*_3_. Assume *a *is hybrid and *b *is its unique child. Since *a *is hybrid it has two parents *q*_1 _and *q*_2_. Choose a normal path from *q*_1 _to *w*_1 _∈ *X *and from *q*_2 _to *w*_2 _∈ *X*. Assume *b *has one normal child *c *and a hybrid child *h *for which there is exactly one other parent *q*. Choose a normal path from *c *to *x *∈ *X*, from *h *to *y *∈ *X*, and from *q *to *z *∈ *X*.

**Figure 7:**
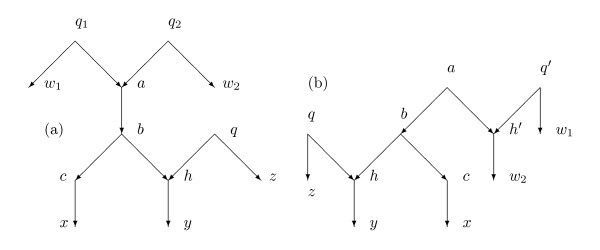
** Case *A*_2_*B*_3_(a)**,** Case *A*_3_*B*_3_(b).**

See Figure 7a. An argument like that for Lemma 4.4 shows that the figure accurately represents what is needed in the argument. In particular, the normal paths from *q*_1 _to *w*_1_, from *q*_2 _to *w*_2_, and from *q *to *x *have no vertex in common. Similarly the paths from *q *to *z*, from *b *to *x*, and from *h *to *y *have no vertex in common.

By Lemma 4.4, *d*(*a*, *x*; *N*) = [*d*(*x*, *w*_1_; *N*) + *d*(*x*, *w*_2_; *N*) *- d*(*w*_1_, *w*_2_; *N*)]/2. In the equiprobable case, by Lemma 4.7, *d*(*b*, *x*; *N*) = *d*(*x*, *y*; *N*) *- d*(*r*, *y*; *N*) + [*d*(*r*, *x*; *N*) + *d*(*r*, *z*; *N*) *- d*(*x*, *z*; *N*)]/2.

In the non-equiprobable case, Lemma 4.9(ii) or 4.9(iii) similarly yields *d*(*b*, *x*; *N*). But *ω*(*a*, *b*) = *d*(*a*, *x*; *N*) *- d*(*b*, *x*; *N*) since the path from *a *to *x *is normal, so subtracting these formulas leads to a formula for *ω*(*a*, *b*).

Case *A*_3_*B*_3_. Assume that *a *has a hybrid child *h*' with other parent *q*'. Choose a normal path from *q*' to *w*_1 _∈ *X *and from *h*' to *w*_2 _∈ *X*. Assume *b *has one normal child *c *and a hybrid child *h *for which there is exactly one other parent *q*. Choose is a normal path from *c *to *x *∈ *X*, from *h *to *y *∈ *X*, and from *q *to *z *∈ *X*.

See Figure 7b. The argument will make two uses of Lemma 4.7 or 4.9, and Figure 7b accurately represents the situation by arguments like those in Lemma 4.4.

In the equiprobable case, by Lemma 4.7, *d*(*a*, *x*; *N*) = *d*(*x*, *w*_2_; *N*)*-d*(*r*, *w*_2_; *N*)+[*d*(*r*, *x*; *N*)+*d*(*r*, *w*_1_; *N*)*-d*(*x*, *w*_1_; *N*)]/2, *d*(*b*, *x*; *N*) = *d*(*x*, *y*; *N*) *- d*(*r*, *y*; *N*) + [*d*(*r*, *x*; *N*) + *d*(*r*, *z*; *N*) *- d*(*x*, *z*; *N*)]/2.

But then *ω*(*a*, *b*) = *d*(*a*, *x*; *N*) *- d*(*b*, *x*; *N*) since the path from *a *to *x *is normal. In the other case, Lemma 4.9(ii) or 4.9(iii) yields *d*(*a*, *x*; *N*) and *d*(*b*, *x*; *N*) and again *ω*(*a*, *b*) is determined. Moreover, Lemma 4.9(i) yields *α*(*a*, *h*') and *α*(*q*, *h*).

Since all 9 cases yield a formula for *ω*(*a*, *b*) and also any relevant probability when *a *is parent to a hybrid and *b *is a normal child of *a*, the proof of the theorem is complete.

**Corollary 4.11**. *Suppose N *= (*V*, *A*, *r*, *X*) *is a normal phylogenetic X-network such that each hybrid vertex has indegree 2 and, if it is not a leaf, outdegree 1. Let n *= |*X*| *and a be the total number of arcs directed into any normal vertex. Then *$a\leq \left(\begin{array}{c} n\\ 2 \end{array}\right)$.

*Proof*. We may assume that the arcs have weights and that each hybrid is equiprobable. Each of the weights *ω*(*u*, *v*) if (*u*, *v*) is an arc directed into a normal vertex *v *is uniquely determined from the $\left(\begin{array}{c} n\\ 2 \end{array}\right)$ linear equations obtained from the $\left(\begin{array}{c} n\\ 2 \end{array}\right)$ distances given by the tree-average distance function. Hence there are at most $\left(\begin{array}{c} n\\ 2 \end{array}\right)$ variables. □

Figure 1 gives an example in which *n *= 4 and there are exactly $\left(\begin{array}{c} 4\\ 2 \end{array}\right)=6$ arcs directed into a normal vertex. Hence the bound in Corollary 4.11 is tight.

## 5 An example

We illustrate the calculations of Section 4 to find the values of the weight function given the network and the tree-average distance. Figure 4 exhibits a phylogenetic *X*-network *N *= (*V*, *A*, *r*, *X*) with *X *= {1, 2, 3, 4, 5, 6, 7, 8, 9, 10, 11} and root 1 which satisfies the hypotheses of Theorem 4.1. Observe that by Corollary 3.4, *N *displays exactly 4 trees, and there are exactly four parent maps. Let *ω *be a weight function on *A *such that *ω*(*a*, *b*) = 0 when *b *is hybrid but *ω*(*a*, *b*) ≥ 0 when *b *is normal. Let *d*(*x*, *y*) = *d*(*x*, *y*; *N*) denote the resulting tree-average distance between *x *and *y *in *X*. Suppose first that we assume equiprobability about the network, so each *α*(*a*, *b*) = 1/2 when *b *is hybrid. There are 24 arcs for which we compute the weights as follows:

First, since 16 and 20 are hybrid, we have *ω*(17, 16) = *ω*(15, 16) = *ω*(21, 20) = *ω*(23, 20) = 0.

By Lemma 4.3(2),

*ω*(19, 8) = [*d*(8, 1) + *d*(7, 8) *- d*(1, 7)]/2, *ω*(19, 7) = [*d*(7, 1) + *d*(7, 8) *- d*(1, 8)]/2, and we similarly find *ω*(14, 3), *ω*(13, 2), and *ω*(22, 10).

By Lemma 4.3(1), *ω*(1, 22) = [*d*(1, 9) + *d*(1, 11) *- d*(9, 11)]/2. By Lemma 4.3(3), *ω*(18, 19) = [*d*(1, 8) + *d*(6, 7) *- d*(1, 6) *- d*(7, 8)]/2, *ω*(12, 13) = [*d*(1, 2) + *d*(11, 3) *- d*(1, 11) *- d*(2, 3)]/2, and we similarly find *ω*(13, 14) and *ω*(22, 12).

By Lemma 4.5, *ω*(20, 18) = [*d*(9, 8) + *d*(11, 6) *- d*(9, 11) *- d*(8, 6)]/2. By Lemma 4.4, *ω*(16, 5) = [*d*(5, 4) + *d*(5, 6) *- d*(4, 6)]/2.

By Lemma 4.7 in the equiprobable case, *ω*(21, 9) = *d*(9, 7) *- d*(1, 7) + [*d*(1, 9) + *d*(1, 11) *- d*(9, 11)]/2, *ω*(23, 11) = *d*(11, 7) *- d*(1, 7) + [*d*(1, 9) + *d*(1, 11) *- d*(9, 11)]/2, and we similarly find *ω*(17, 6) and *ω*(15, 4).

By Lemma 4.3(2), *d*(18, 6) = [*d*(6, 1) + *d*(6, 7) *- d*(1, 7)]/2. But then *ω*(18, 17) = *d*(18, 6) *- ω*(17, 6).

Similarly by Lemma 4.2(2) *d*(14, 4) = [*d*(4, 1) + *d*(4, 3) *- d*(1, 3)]/2 and then *ω*(14, 15) = *d*(14, 4) *- ω*(15, 4).

Similarly by Lemma 4.3(2) *d*(12, 11) = [*d*(11, 1) + *d*(11, 2) *- d*(1, 2)]/2 and then *ω*(12, 23) = *d*(12, 11) *- ω*(23, 11).

Finally, *d*(10, 9) is known since 10 ∈ *X*, so *ω*(10, 21) = *d*(10, 9) *- ω*(21, 9). This concludes the calculation of all the weights for *N *in the equiprobable case. Note that in several of these calculations, there were alternative choices possible. For example, we also have *ω*(22, 12) = [*d*(1, 4) + *d*(9, 11) *- d*(1, 9) *- d*(4, 11)]/2.

The general case where we do not assume equiprobability proceeds in a similar manner, different from the above only in the use of Lemma 4.9 in place of Lemma 4.7. We compute *ω*(21, 9), *ω*(23, 11), *α*(21, 20), and *α*(23, 20) using Lemma 4.9 with *x*_1 _= 9, *x*_2 _= 11, *x*_3 _= 2, and *y *= 7. We compute *ω*(17, 6), *ω*(15, 4), *α*(17, 16), and *α*(15, 16) using Lemma 4.9 with *x*_1 _= 6, *x*_2 _= 4, *x*_3 _= 3, *y *= 5.

## 6 Extensions

Theorem 4.1 applies only to normal phylogenetic networks for which the indegree of each hybrid vertex is 2. It would be interesting to see whether the same results are true without the restriction on the indegree of a hybrid vertex. Whereas I have verified this for several individual networks with vertices of indegree 3 or 4, I do not have a general proof.

In the event of a true hybridization between two sexual species, it is plausible to assume that the indegree is 2 and that each parent contributes approximately equally. Hence in this case it is plausible that we would obtain the tree-average distance utilized in Theorem 4.1. Nevertheless, backcrossing of the hybrid *h *with one of the parental species *q*_1 _could easily increase the fraction of the genome of *q*_1 _in *h*, changing it from 50%. Similarly, if the reticulation is actually a horizontal gene transfer, common between bacteria, then there is no guarantee that the sources contribute approximately equally. Hence the occurrence of probabilities different from 1/2 seems likely.

## Competing interests

The author declares that they have no competing interests.
